# A multifunctional injectable microsphere with enhanced near-infrared photo-antibacterial, ROS scavenging, and anti-inflammatory properties for periodontitis treatment

**DOI:** 10.7150/thno.107793

**Published:** 2025-03-03

**Authors:** Ziliang Xiu, Yujing Zhu, Xiaofeng Li, Xiaoxi Jiang, Yunru Feng, Li He, Chunhui Li, Rui Cai, Gang Tao

**Affiliations:** 1 Oral & Maxillofacial Reconstruction and Regeneration of Luzhou Key Laboratory, The Affiliated Stomatological Hospital, Southwest Medical University, Luzhou 646000, China.; 2 Department of Periodontics & Oral Mucosal Diseases, The Affiliated Stomatological Hospital, Southwest Medical University, Luzhou 646000, China.; 3 Department of Oral Medicine, Deyang Stomatological Hospital, Deyang 618000, China.; 4 Institute of Stomatology, Southwest Medical University, Luzhou 646000, China.

**Keywords:** Periodontitis, Multifunctional microsphere, Photothermal antibacterial, ROS scavenging, Anti-inflammation

## Abstract

**Background:** Pathogenic bacteria and activated immune cells in periodontal tissues continuously generate reactive oxygen species (ROS) and inflammatory mediators, creating an oxidative stress microenvironment that causes damage to cells and tissues. Antimicrobial strategies and ROS scavenging are crucial for treating periodontitis.

**Methods:** This study encapsulated iron-curcumin nanoparticles (Fe-Cur NPs) within GelMA microspheres using microfluidic technology. The microsphere surface was then modified with a polydopamine (PDA) layer, and minocycline hydrochloride (MH) was attached to it to form multifunctional composite microspheres (GM@Fe-Cur/PDA/MH).

**Results:** The GM@Fe-Cur/PDA/MH microspheres exhibited excellent adhesion, allowing them to remain in periodontal pockets for prolonged periods to maintain their functionality. Benefiting from the photothermal properties of PDA combined with MH, these microspheres effectively kill bacteria and mitigate excessive immune responses. Additionally, they regulate the M1/M2 polarization of macrophages, suppress the expression of inflammatory factors, and promote osteogenic protein expression under oxidative stress conditions *in vitro*. In a rat model of periodontitis, GM@Fe-Cur/PDA/MH effectively controlled inflammation progression and reduced alveolar bone loss. Further studies indicated that GM@Fe-Cur/PDA/MH may promote ROS scavenging by modulating the nuclear factor erythroid 2-related factor 2 (Nrf2) signaling pathway while inhibiting the activation of the nod-like receptor protein-3 (NLRP3) inflammasome.

**Conclusions:** In summary, GM@Fe-Cur/PDA/MH represents an effective therapeutic approach that integrates antibacterial, antioxidant, anti-inflammatory, and bone loss mitigation properties, demonstrating significant potential for application in the treatment of periodontitis.

## Introduction

Periodontitis is a chronic inflammatory disease driven by an imbalance in the local microbiome and dysregulation of the host immune response, affecting over 1 billion people worldwide 1. The pathogenic bacteria colonizing dental plaque stimulate the host immune system, generating high levels of reactive oxygen species (ROS) and inflammatory mediators, including tumor necrosis factor-alpha (TNF-α) and interleukin-1 beta (IL-1β) 2, 3. This inflammatory cascade leads to the destruction of periodontal tissues, resorption of the alveolar bone, and, ultimately, tooth loss 4. In addition, periodontitis is closely associated with systemic diseases like cardiovascular diseases and diabetes mellitus, placing a heavy burden on both patients and the public health system 5. Current treatment strategies, primarily mechanical debridement techniques like scaling and root planning, can effectively remove plaque and calculus. However, they have limitations in accessing deep periodontal pockets and irregular root surfaces, often leading to disease recurrence 6. Therefore, there is an urgent need to develop materials that can adapt to the complex periodontal structure and possess antibacterial, ROS-scavenging, and anti-inflammatory properties for more effective periodontitis treatment 7.

In the early stages of periodontitis, macrophages engulf pathogens and secrete pro-inflammatory cytokines, such as TNF-α, to recruit immune cells to the infection site, enhancing the local immune response 8. However, the overactivation and aggregation of macrophages also result in excessive ROS production, including superoxide anion (O^2-^) and hydrogen peroxide (H_2_O_2_), triggering oxidative stress and significant damage to periodontal tissues 9. As inflammation progresses, macrophages transform from an M1 to an M2 phenotype 10. M1 macrophages exacerbate inflammation and bone resorption by secreting pro-inflammatory cytokines like TNF-α and IL-1β, whereas M2 macrophages promote inflammation resolution and tissue repair by producing anti-inflammatory cytokines, such as interleukin-10 (IL-10) 11. Therefore, effective treatment of periodontitis not only requires the elimination of pathogens and excessive ROS but also the regulation of macrophage polarization from the pro-inflammatory M1 phenotype to the reparative M2 phenotype. This shift is crucial for promoting the repair and regeneration of periodontal tissues.

Curcumin, a natural polyphenolic compound, has been extensively studied for its potent anti-inflammatory and antioxidant properties 12. Although clinical trials have demonstrated that topical application of curcumin effectively alleviates periodontal inflammation, its poor bioavailability and low solubility limit its therapeutic potential 13. Therefore, enhancing the bioavailability of curcumin is a critical strategy for its effective use in periodontitis treatment. In recent years, nanomedicine development has provided novel solutions to improve curcumin's bioavailability 14. Combining metal ions with organic ligands makes it possible to form nanoscale coordination polymers with rich properties, stable structures, and ease of synthesis 15, 16. In this study, we synthesized fully water-soluble iron-curcumin coordination nanoparticles (Fe-Cur NPs) for the anti-inflammatory needs of periodontitis treatment by combining iron ions with curcumin. Nevertheless, the direct application of Fe-Cur NPs to periodontal sites still presents challenges, including rapid drug loss and difficulty in sustaining long-term effects.

To address these issues, we designed a microsphere-based drug delivery system. Microspheres are ideal carriers that can adapt to irregular tissue structures and continuously release drugs during degradation 17. Through microfluidic technology, we precisely controlled the flow rates of the oil and water phases to produce microspheres with uniform sizes in large quantities 18. Gelatin methacryloyl (GelMA), known for its excellent biocompatibility and biodegradability, was employed for microsphere fabrication 19. However, GelMA microspheres (GM) generally lack adhesion properties, leading to short drug retention times and reduced efficacy. Polydopamine (PDA), inspired by mussel adhesive proteins, has excellent biocompatibility and adhesion capabilities. Modifying GM with PDA significantly enhances their adhesion and retention in periodontal tissues 20. Furthermore, PDA exhibits high near-infrared (NIR) light absorption and remarkable photothermal conversion capabilities, enabling the generation of elevated temperatures through NIR irradiation, effectively eradicating bacteria 21. However, effective photothermal antimicrobial treatments typically require high temperatures (>60 °C), which may result in thermal injury to healthy tissue 22. Minocycline hydrochloride (MH) is a preferred antibiotic for treating periodontitis. By combining photothermal therapy with antibiotics, we can overcome the limitations of low-temperature antibacterial action while reducing the risk of antibiotic resistance, further enhancing the antibacterial effect 23, 24.

**Scheme 1:**
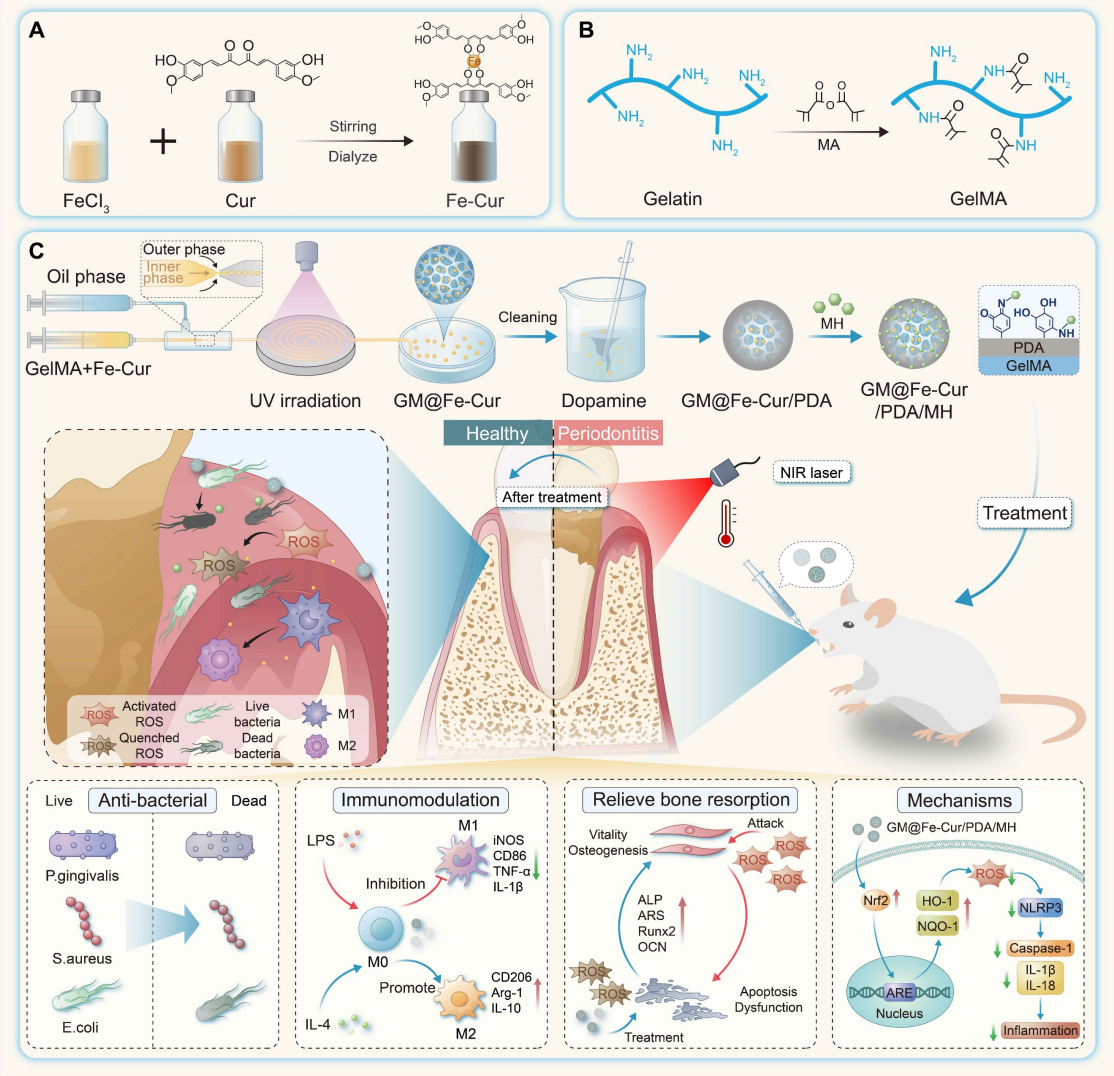
** Schematic illustration of the fabrication process of GM@Fe-Cur/PDA/MH microspheres and treatment of periodontitis. (A)** Schematic of the synthesis of Fe-Cur NPs. **(B)** Schematic diagram of chemical modification of gelatin. **(C)** Schematic diagram of the GM@Fe-Cur/PDA/MH synthesis process and injection into periodontal pockets for the periodontitis of treatment.

In this study, we developed GM@Fe-Cur/PDA/MH composite microspheres to address the antibacterial and antioxidant needs in treating periodontitis. First, combining curcumin with iron ions overcame the limitations of curcumin's low bioavailability and poor water solubility. Next, we utilized microfluidic technology to prepare GM of uniform size, encapsulating Fe-Cur NPs to achieve sustained release. Subsequently, the surface of the microspheres was modified with a PDA layer through the self-polymerization of dopamine (DA), and MH was further loaded, endowing the microspheres with excellent adhesion, photothermal conversion properties, and potent antibacterial activity **(Scheme 1)**. *In vitro* experiments verified the antioxidant activity, photothermal performance, and antibacterial efficacy of the composite microspheres while also assessing their immunomodulatory effects on macrophages and their influence on osteogenic differentiation of human periodontal ligament stem cells (hPDLSCs) under oxidative stress conditions. Furthermore, the practical application of GM@Fe-Cur/PDA/MH was validated using a rat model of periodontitis. Finally, additional mechanistic studies indicated that the nuclear factor erythroid 2-related factor 2 (Nrf2) signaling pathway and nod-like receptor protein-3 (NLRP3) inflammasome may play significant roles in the capacity of GM@Fe-Cur/PDA/MH to scavenge ROS and alleviate inflammatory responses. Collectively, these findings suggest that GM@Fe-Cur/PDA/MH holds promise in overcoming the limitations of current periodontitis treatments, offering an effective strategy for antibacterial, antioxidant, and immunomodulatory therapies.

## Materials and Methods

### Materials

Ferric (III) trichloride hexahydrate (FeCl_3_·6H_2_O) and polyvinylpyrrolidone (PVP) were purchased from Aladdin (Shanghai, China). Curcumin was purchased from J&K Scientific (Beijing, China). Dopamine hydrochloride was purchased from Macklin Biochemical Technology (Shanghai, China). Minocycline hydrochloride (MH) was purchased from Meilun Biotechnology (Dalian, China). Artificial saliva was purchased from Chuangfeng Automation Technology (Guangzhou, China). Alpha-modified eagle medium (α-MEM), Dulbecco's Modified Eagle Medium (DMEM), 0.25% trypsin-EDTA, and fetal bovine serum (FBS) were purchased from Gibco (MD, USA). Cell Counting Kit-8, 1% penicillin-streptomycin, and 4% paraformaldehyde were purchased from Beyotime (Beijing, China). Live/Dead bacterial staining kits were purchased from Thermo Fisher Scientific (MA, USA). All other materials and reagents for this study were applied directly without further purification.

### Synthesis and characterization of Fe-Cur NPs

First, 66 mg of PVP was dissolved in 5 mL of methanol. Subsequently, 20 mg of FeCl_3_·6H_2_O, dissolved in 1 mL of methanol, was added dropwise under continuous stirring. Then, 10 mg of curcumin, dissolved in 1 mL of methanol, was slowly added dropwise to the mixture, stirring for 3 h. The obtained solution was then dialyzed in deionized water overnight. After collection, the solution was stored at 4 °C for further experiments.

Transmission electron microscopy (TEM, JEM-2100, Tokyo, Japan) was used to observe the size distribution of Fe-Cur NPs. The ultraviolet-visible-near-infrared (UV-vis-NIR) spectra of curcumin and Fe-Cur NPs were recorded using a UV-vis spectrophotometer (TU-1810, Shanghai, China). Fourier-transform infrared (FTIR) spectra of curcumin and Fe-Cur NPs were measured by FTIR spectrometer (WQF-530, Beijing, China). Additionally, X-ray photoelectron spectroscopy (XPS, AXIS SUPRA+, Nagoya, Japan) was used to analyze the chemical states of the elements present in Fe-Cur NPs.

### Preparation and characterization of GM@Fe-Cur/PDA/MH

GelMA-based microspheres were fabricated using microfluidic technology. The aqueous phase (5 wt.% GelMA solution with 0.5 wt.% photoinitiators) and the oil phase (10 wt.% Span 80 dissolved in isopropyl myristate) were injected into two inlets of the microfluidic device. Adjusting the flow rates of the two phases, the shear force was controlled to generate droplets of the desired size. The formed microdroplets then received sufficient UV cross-linking in a sufficiently long tube to form microspheres after curing, which were then collected and washed multiple times with 75% ethanol to remove surfactant and oil residues. Subsequently, the Fe-Cur NPs solution was added to the aqueous phase at a final concentration of 200 μg/mL while keeping the concentrations of GelMA solution and photoinitiators unchanged to synthesize GM@Fe-Cur microspheres. The cleaned GM@Fe-Cur microspheres were then immersed in a simulated marine environment (10 mM Tris, 4 mg/mL DA, pH 8.5) and stirred for about 12 h to form a PDA layer. After washing with PBS to remove excess DA, GM@Fe-Cur/PDA microspheres were obtained. To prepare GM@Fe-Cur/PDA/MH microspheres, a 0.5 mg/mL solution of MH was used, with the pH adjusted to neutral. The GM@Fe-Cur/PDA microspheres were added to this solution and stirred slowly for about 2 h. Following a final PBS wash, GM@Fe-Cur/PDA/MH microspheres were obtained.

The morphology and size of GM, GM@Fe-Cur, GM@Fe-Cur/PDA, GM@Fe-Cur/PDA/MH were observed using an optical microscopy, and the diameters were statistically analyzed using Nano Measurer v1.2.5 software (with more than 200 samples). The microspheres were dried using a freeze dryer (Sientz-12N, Ningbo, China). Their surface morphology was observed by SEM (Zeiss Sigma300, Germany). Energy-dispersive spectroscopy (EDS) was employed to analyze the elemental distribution of the microspheres.

### Adhesion, injectability, and degradation behavior of GM@Fe-Cur/PDA/MH

In brief, GM@Fe-Cur/PDA/MH adhered to the surfaces of human-isolated teeth, metal, glass, and plastic to preliminarily evaluate their adhesion properties. Subsequently, isolated rat maxillary gingival tissues were used to assess the adhesion ability of GM@Fe-Cur/PDA/MH to biological tissues. Finally, after attaching GM@Fe-Cur/PDA/MH to human-isolated teeth, a specific flow rate (0.3 mL/min, 0.6 mL/min, and 0.9 mL/min) of water was applied and continued for a certain period. The remaining adhesion area of the microspheres at different time points was observed and measured using ImageJ software to evaluate the microsphere retention rate under fluid flow. Injectability was tested by placing GM@Fe-Cur/PDA/MH in a white pipette tip with a volume range of 0.5-10 μL, injecting it, and observing the result.

To investigate the degradation performance of the microspheres, GM@Fe-Cur/PDA/MH were separately immersed in 500 μL of PBS solution, 500 μL artificial saliva, or 500 μL artificial saliva + 100 ng/mL lipopolysaccharide (LPS), and incubated at 37 °C, 80 rpm. Fresh artificial saliva and LPS were replaced daily, while PBS was refreshed every three days to maintain the stability of the solution. At the designated time points, morphological changes in the microspheres were observed and recorded using an optical microscope.

### *In vitro* drug release study

The loading content (LC) and encapsulation efficiency (EE) of MH in GM@Fe-Cur/PDA/MH were determined using the absorbance method. The formulas for calculating LC and EE are as follows:

LC (%) = (Wmh - CsV)/Wms × 100%

EE (%) = (Wmh - CsV)/Wmh × 100%

Where Wmh is the initial total weight of MH, Cs is the concentration of MH in the supernatant after loading, V is the volume of the supernatant after loading, and Wms is the total weight of GM@Fe-Cur/PDA/MH.

Additionally, GM@Fe-Cur/PDA/MH was freeze-dried and then placed in 1 mL of PBS solution and incubated on a shaker (37 °C, 80 rpm) for 48 h. The concentration of Fe-Cur NPs in the release medium was measured and compared to the initial concentration of Fe-Cur NPs in the GelMA aqueous phase to calculate the loading rate (LR) of Fe-Cur NPs. The formula for calculating LR is as follows:

LR (%) = C_2_V_2_/C_1_V_1_ × 100%

Where C_2_ is the concentration of Fe-Cur NPs in the supernatant after incubating the freeze-dried GM@Fe-Cur/PDA/MH for 48 h, V_2_ is the volume of the supernatant after incubation, C_1_ is the initial concentration of Fe-Cur NPs in the GelMA aqueous phase, and V_1_ is the volume of the initial GelMA aqueous phase.

Subsequently, GM@Fe-Cur/PDA/MH was placed in 5 mL of PBS, artificial saliva, and artificial saliva + 100 ng/mL LPS to simulate normal physiological conditions, a non-inflammatory oral environment, and an inflammatory oral environment, respectively. The samples were incubated at 37 °C, 80 rpm. At designated time points, 100 μL or 1 mL of solution was sampled for absorbance testing, and fresh corresponding solution of the same volume was added to maintain the stability of the solution volume throughout the release process. The cumulative drug release was calculated using the following formula:

Drug Release (%) = 
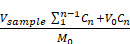


Where M_0_ represents the initial amount of MH or Fe-Cur NPs in the solution (μg), V_0_ is the total volume of the release system (5 mL), V_sample_ is the volume taken for each sample (100 μL for MH and 1 mL for Fe-Cur NPs), and C_n_ is the drug concentration in the release medium at the n-th time point.

### Culture and identification of hPDLSCs

This study was approved by the Ethics Committee of the Affiliated Stomatological Hospital of Southwest Medical University (Document No. 20220819003). The samples were obtained from healthy teeth extracted for orthodontic treatment at the Affiliated Stomatological Hospital. After obtaining informed consent, the extracted teeth were collected, and periodontal ligament tissues were separated from the root surfaces using a sterile blade in an ultra-clean bench. After washing and centrifugation, the isolated periodontal ligament tissues were cultured in α-MEM medium containing 10% FBS and 1% penicillin-streptomycin in a 5% CO_2_ atmosphere at 37 °C. The medium was changed every two days, and the cells were passaged when they reached 70%-80% confluence. For subsequent experiments, P3-P6 cells were used.

At P3, cells were treated with 0.25% Trypsin-EDTA (1X), washed, and centrifuged. Then, hPDLSCs (5 × 10^5^ cells/mL, 1 mL) were incubated with human antibodies against CD31 (FITC), CD45 (FITC), CD90 (PE), and CD105 (FITC) (Biolegend, CA, USA). Surface markers of the stem cells were detected using flow cytometry (FACSCalibur, CA, USA). The data were analyzed with WinMDI 2.8 (Scripps Institute, West Lafayette, IN, USA).

### Biocompatibility of GM@Fe-Cur/PDA/MH

The hemocompatibility of the microspheres was evaluated by co-incubating them with rabbit erythrocytes. Rabbit blood was mixed with sodium citrate and centrifuged for 10 min to discard the supernatant. The erythrocytes were washed three times with PBS and centrifuged again, followed by dilution to a final concentration of 10% (v/v). Next, 50 mg of microspheres were gently mixed with the erythrocyte solution and incubated at 37 °C for 60 min. After centrifugation, the supernatant was collected, and the optical density (OD) value at 545 nm was measured using a microplate reader (Tecan Infinite M200-Pro, China). 0.1% Triton X-100 was used as the positive control, and PBS as the negative control. The hemolysis ratio of the microspheres was calculated using the following formula:

Hemolysis ratio (%) = [(ODm-ODp)/(ODb-ODp)] × 100%

Where ODb is the OD value of 0.1% TritonX-100 positive control group, ODp is the OD value of the PBS negative control group, and ODm is the OD value of the microsphere group.

The cytocompatibility of the microspheres was assessed using live/dead cell staining method and cell counting kit (CCK-8). Briefly, sterilized lyophilized microspheres were incubated at a concentration of 1 mg/mL in α-MEM or DMEM culture medium at 37 °C on a horizontal shaker at 80 rpm for 48 h to prepare the microsphere extracts. hPDLSCs and RAW264.7 cells were seeded into 96-well plates and cultured at 37 °C in a 5% CO_2_ atmosphere. hPDLSCs were cultured in α-MEM medium and RAW264.7 cells in DMEM medium. After 24 h of initial culture, the medium was replaced with the microsphere extracts. On days 1, 3, and 5, CCK-8 reagent was added to the wells and incubated at 37 °C for 2 h in the dark. Absorbance at 450 nm was then measured using a microplate reader. In addition, live/dead cell staining was performed at the same time points. Following a 15-min incubation period in the dark at 37 °C, the cells were observed using a fluorescence microscope (DMI8, Leica, Germany).

### *In vitro* antioxidant properties of GM@Fe-Cur/PDA/MH

The *in vitro* antioxidant capacity of the composite microspheres was assessed using various probes, including 1,1-diphenyl-2-picrylhydrazyl radical (DPPH·), 2,2'-azinobis (3-ethylbenzthiazoline-6-sulfonate) radical (ABTS+·), and 2-Phenyl-4,4,5,5-tetramethylimidazoline-1-oxyl-3-oxide (PTIO·). Mix 50 mg of microspheres with 1.5 mL of extraction solution, then centrifuge the mixture at 10,000 rpm for 10 min at room temperature. Carefully collect the supernatant and keep it on ice. Then, incubate the supernatant with the above probe working solution for 30 min in the dark. Observe and record color changes in the solutions. The absorbance values of the solutions at 515 nm (DPPH·), 405 nm (ABTS+·), and 557 nm (PTIO·) were determined using a UV-vis spectrophotometer. The free radical scavenging rate was calculated using the following formula:

Positive control scavenging rate (%) = [(Ac-Ap)/Ac] × 100%

Sample scavenging rate (%) = [Ac-(Am-Am')]/Ac × 100%

Where Ac represents the absorbance of the free radical working solution blank control, Ap is the absorbance of the positive control vitamin C (Vc), Am is the absorbance of the microspheres after incubation with the working solution, and Am' is the absorbance of the microspheres co-incubated with the working solution solvent.

### Intracellular ROS scavenging activity

To further investigate the antioxidant properties of composite microspheres, the intracellular ROS scavenging ability was evaluated using DCFH-DA (Solarbio, China). RAW264.7 cells (5 × 10^4^ cells/mL, 500 μL) were seeded in 24-well plates and cultured in DMEM medium for 24 h. Subsequently, the medium was replaced with microsphere extracts, and incubation continued for another 24 h. The cells were then stimulated with H_2_O_2_ (100 μM) for 2 h, followed by the addition of DCFH-DA (10 μM). The incubation was carried out for 20 min at 37 °C in the dark. The results were observed and recorded using a fluorescence microscope, and fluorescence intensity was quantitatively analyzed with ImageJ software.

The protective effect of composite microspheres on hPDLSCs and RAW264.7 cells under oxidative stress conditions was assessed through live/dead cell staining and CCK-8 assays. Both hPDLSCs and RAW264.7 cells (5 × 10^4^ cells/mL, 500 μL) were seeded in 24-well plates and cultured in α-MEM and DMEM medium, respectively, for 24 h. After the initial culture period, the medium was replaced with microsphere extracts, and cells were cultured for an additional 24 h. Oxidative stress was induced by adding 150 μM H_2_O_2_ for 2 h. The cells were then incubated with live/dead cell staining solution at 37 °C for 20 min in the dark, followed by fluorescence microscopy observation. Cell viability was further quantified using the CCK-8 assay.

### Photothermal properties of GM@Fe-Cur/PDA/MH

The photothermal properties of the composite microspheres were evaluated under NIR irradiation. Place 50 mg of GM@Fe-Cur/PDA/MH in a centrifuge tube containing 1.5 mL of PBS and irradiate it for 6 min under NIR light at different power levels (0.5 W/cm^2^, 1 W/cm^2^, and 1.5 W/cm^2^) at a distance of approximately 10 cm. The temperature changes of the microspheres were monitored and recorded using a thermography camera (E8-XT, FLIR, OR, USA). After determining the optimal power, the temperature changes of different microspheres were further tested at this power. Finally, the photothermal stability of GM@Fe-Cur/PDA/MH under 808 nm NIR (1 W/cm^2^) irradiation was verified by four laser on/off cycles.

### *In vitro* antibacterial tests

The oral environment is populated by a diverse array of bacteria, and *Staphylococcus aureus* (*S. aureus*), *Escherichia coli* (*E. coli*), and *Porphyromonas gingivalis* (*P. gingivalis*) were selected to test the *in vitro* antimicrobial properties of composite microspheres. Firstly, *S. aureus* and *E. coli* were cultured in Luria-Bertani (LB) liquid medium, and bacterial suspension (1.0 × 10^6^ CFU/mL) was prepared. Microspheres (20 mg) were prepared into 100 µL of injectable suspension using PBS. Subsequently, 500 µL of bacterial suspension was mixed with 100 µL of PBS, GM, GM@Fe-Cur, GM@Fe-Cur/PDA, and GM@Fe-Cur/PDA/MH. One group was irradiated with 808 nm NIR (1.0 W/cm^2^) for 5 min, and another group was not irradiated. The mixtures were co-incubated at 37 °C for 2 h. Subsequently, a total of 100 μL of co-culture was spread onto LB solid medium, and colony counts were recorded after incubation for 12 h at 37 °C. The colony formation counting assay for *P. gingivalis* was conducted as previously described, except that *P. gingivalis* was cultured in Brain Heart Infusion (BHI) liquid medium and plated on blood agar, followed by incubation under anaerobic conditions.

In addition, the bacterial suspension after 2 h of co-culture, as described above, was collected, centrifuged at 10,000 rpm for 10 min, incubated with bacterial live/dead fluorescent dye for 20 min in the dark, and then visualized under a fluorescence microscope.

### Immunomodulatory effect of GM@Fe-Cur/PDA/MH on RAW264.7 cells

Flow cytometry was initially used to detect macrophage phenotype. RAW264.7 cells (5 × 10^4^ cells/mL, 2 mL) were seeded into 6-well plates and divided into six groups: 1) Control group. 2) LPS or interleukin-4 (IL-4) group. 3) GM group. 4) GM@Fe-Cur group. 5) GM@Fe-Cur/PDA group. 6) GM@Fe-Cur/PDA/MH group. After 24 h of incubation in DMEM medium, cells in the LPS or IL-4 group and different microsphere groups were treated with LPS (100 ng/mL, Sigma, USA) or IL-4 (20 ng/mL, Pepro Tech, USA) for a further 24 h. The LPS or IL-4 group medium was then replaced with a new DMEM medium and continued to culture. Meanwhile, the different microsphere groups were cultured with microsphere extracts, and the control group was cultured under normal conditions without additional treatment. After continuing the culture for 48 h, the cells were subjected to enzymatic digestion, centrifugation (800 rpm, 3 min), and washes with PBS. Subsequently, the cells were incubated with anti-CD86 (105005, Biolgend) and anti-CD206 (147105, Biolgend) antibodies for 20 min in the dark. Finally, the cells were analyzed using flow cytometry (ACEA NovoCyte^TM^ 2070R, California, USA).

Immunofluorescence staining was used to identify the surface markers of distinct macrophage phenotypes, which were processed in a manner analogous to flow cytometry. The incubation was continued for 48 h using microsphere extracts, after which the samples were fixed with 4% paraformaldehyde for 15 min, followed by adding 0.5% Triton X-100 for 15 min. After this, 5% goat serum was added and incubated for 1.5 h at 37 °C. The antibodies anti-iNOS (1: 100, Proteintech, China) or anti-CD206 (1:100, Proteintech, China) were incubated overnight at 4 °C in the dark. After overnight incubation, the cells were treated with Alexa Fluor 647 Conjugate (1:200, Cell Signaling Technology, USA) for 1 h, followed by DAPI staining for 20 min. The intensity of the red fluorescence was observed using confocal microscopy (SOPTOP IRX50, Sunny Optical Technology, China), and the images were analyzed with ImageJ software.

Real-time quantitative reverse transcription PCR (qRT-PCR) was employed to assess the mRNA expression levels of inflammation-related genes (TNF-α, IL-1β, IL-10, and Arg-1) in RAW264.7 cells. Macrophages (5 × 10^4^ cells/mL, 2 mL) were seeded in 6-well plates and stimulated with LPS, following the same grouping and treatment protocol as used in flow cytometry. Total RNA was isolated using the SteadyPure Quick RNA Extraction Kit (Accurate Biotechnology, Hunan, China) and subsequently reverse transcribed into cDNA with the Evo M-MLV RT Kit (Accurate Biotechnology, Hunan, China). The qRT-PCR analysis was then carried out on the cDNA samples utilizing the CFX96 Touch Real-Time PCR Detection System (Bio-Rad, California, USA). The sequences of the qPCR primers are provided in Table S1, with β-actin serving as the reference gene. The 2^-ΔΔCt^ method was utilized to compare mRNA expression levels between different groups.

### Effect of GM@Fe-Cur/PDA/MH on osteogenic differentiation of hPDLSCs under oxidative stress environment

The hPDLSCs of P3-P6 were collected, and the cells (3 × 10^4^ cells/mL, 1 mL) were inoculated into 12-well plates. The cells were initially cultured in α-MEM medium for 24 h. After this period, the medium was replaced with microsphere extracts, and the cells were cultured for an additional 24 h. Subsequently, the cells were stimulated by H_2_O_2_ (150 μM) for 2 h. After removing the microsphere extracts, the cells were treated with an osteogenic induction solution (1% penicillin-streptomycin, 10% FBS, 5 mg Vc, 216 mg β-glycerophosphate, and 4 μL dexamethasone in 100 mL α-MEM medium). Following a 7-day osteogenic induction period, the cells were rinsed with PBS, fixed in 4% paraformaldehyde for 15 min, and stained for alkaline phosphatase (ALP) using a BCIP/NBT ALP staining kit (Beyotime, China). In a parallel experiment, cells were seeded and treated similarly, but following 14 days of osteogenic induction, the cells were washed, fixed, and stained with 0.1 % alizarin red S (ARS, Solarbio, China) to evaluate mineralization.

For immunofluorescence staining, cells were seeded onto coverslips in 24-well plates (3 × 10^4^ cells/mL, 500 μL) and cultured in α-MEM medium for 24 h. The medium was then replaced with microsphere extracts for an additional 24-h incubation. Cells were subsequently stimulated with H_2_O_2_ (150 μM) for 2 h. Following this, the microsphere extracts were discarded, and an osteogenic induction medium was added. Cells were then incubated for 7 or 14 days. At the end of the incubation period, cells were fixed with 4% paraformaldehyde for 15 min, treated with 0.5% Triton X-100 for 15 min, and blocked with 5% goat serum for 1.5 h at 37 °C. Cells were then incubated overnight at 4 °C in the dark with anti-Runx2 (1:100, Cell Signaling Technology, USA) or anti-OCN (1:100, Proteintech, China). Followed by a 1-h incubation with Alexa Fluor 647 conjugate, the cytoskeleton and nuclei were incubated with FITC (1:100) and DAPI in the dark for 1.5 h and 20 min, respectively. Finally, the fluorescence intensity was observed using a confocal microscope (BX53, Olympus, Japan), and images were analyzed with ImageJ software.

### Establishment of rat periodontitis model

The experimental subjects were male SD rats of 5-6 weeks of age. The animal protocol was approved by the Ethics Committee of Southwest Medical University (Document No. 20240418-009). SD rats were first fed in a standard light-dark cycle for 1 week to acclimatize to the environment. Subsequently, the rats were randomly divided into 6 groups (n=6), including 1) Control group: no treatment. 2) Periodontitis group: ligation and local injection of bacterial suspension. 3) GM group: periodontitis treated with GM. 4) GM@Fe-Cur group: periodontitis treated with GM@Fe-Cur. 5) GM@Fe-Cur/PDA/MH group: periodontitis treated with GM@Fe-Cur/PDA/MH. 6) GM@Fe-Cur/PDA/MH+NIR group: periodontitis treated with GM@Fe-Cur/PDA/MH and NIR light irradiation.

Rats were anesthetized by inhalation of isoflurane (2%, 100% oxygen), and 2-0 orthodontic ligature wires were then placed in the cervical region of the maxillary first molar. A suspension of *P. gingivalis* (10 μL) was injected around the ligature wires every 3 days. After 2 weeks, the ligature wires were removed, and the corresponding treatments were initiated. The control group received no treatment. The periodontitis group was injected with 10 µL of PBS at the ligature site every 3 days. In the microsphere groups, 10 µL of PBS solution containing approximately 0.33 mg of microspheres was injected at the ligature site every 3 days. The GM@Fe-Cur/PDA/MH+NIR group received 5 min of NIR irradiation treatment at a power intensity of 1 W/cm^2^, and the temperature in the periodontal pockets was monitored using a thermal imager.

After 2 weeks of microsphere treatment, rats were anesthetized with isoflurane. DCFH-DA (1.8 mg/kg) was injected into the cervical gingiva of the maxillary first molar. Following a 20-min incubation, fluorescence imaging and intensity analysis were conducted using an *in vivo* small animal imaging system (ABL X6, Tanon, China) at an excitation wavelength of 490 nm to evaluate ROS clearance of microspheres in the rats. Additionally, the ability of microspheres to reduce intracellular ROS *in vitro* was assessed. RAW264.7 cells (5 × 10^4^ cells/mL, 1 mL) were seeded in 12-well plates and cultured in DMEM medium for 24 h. After replacing the medium with microsphere extracts and incubating for another 24 h, the cells were stimulated with LPS (100 ng/mL) for 2 h. DCFH-DA (10 μM) was added and incubated at 37 °C in the dark for 20 min. Fluorescence imaging at 490 nm and subsequent fluorescence intensity analysis were performed.

### Micro-CT analysis

At weeks 2 and 4, following microsphere treatment, the rats were euthanized with an overdose of isoflurane. Maxillary bone samples were then collected, thoroughly washed with PBS, and fixed in 4% paraformaldehyde. These samples were scanned at a resolution of 3.8 μm (nanoVoxel-100, Tianjin, China). 3D model reconstruction and 2D image analysis were performed using RadiAnt DICOM Viewer (2023.1) software.

### Histological analysis

Maxillary bone samples were collected at weeks 2 and 4. The samples were initially fixed in 4% paraformaldehyde and decalcified in a 10% ethylenediaminetetraacetic acid (EDTA) solution for 1 month. Subsequently, the samples were embedded in paraffin and sectioned into 4 μm slices, which were mounted on slides. The tissue sections underwent hematoxylin-eosin (H&E) staining and Masson's trichrome staining, then scanned using a digital pathology slide scanner (KF-PRO-002, China). The sections were then subjected to immunofluorescence staining (markers: Runx2, OCN, iNOS, CD206, Nrf2, NLRP3) and immunohistochemistry staining (markers: TNF-α, IL-1β, IL-6). The stained images were analyzed using ImageJ software.

### Anti-inflammatory and antioxidant mechanisms of GM@Fe-Cur/PDA/MH

qRT-PCR was employed to assess the mRNA expression levels of antioxidant gene NQO-1 and inflammatory cytokines Caspase-1 and IL-18 in RAW264.7 cells. Macrophages (5 × 10^4^ cells/mL, 2 mL) were seeded in 6-well plates and stimulated with LPS, with grouping and treatment processes consistent with flow cytometry experiments. Total RNA was extracted using a kit, and the RNA was reverse-transcribed into cDNA, followed by qRT-PCR analysis of the cDNA samples. The qPCR primer sequences are provided in Table S1, with β-actin serving as the reference gene. The 2^-ΔΔCt^ method evaluated the mRNA expression levels across different treatment groups.

Western blot was conducted to detect antioxidant proteins (Nrf2 and HO-1), inflammasome component NLRP3, and β-actin for protein expression analysis. Macrophages were seeded and treated as in the qRT-PCR experiments. The cells were lysed in RIPA lysis buffer (Epizyme Biotech, Shanghai, China) and incubated on ice for 10 min. The lysate was then sonicated and centrifuged at 12,000 rpm for 10 min at 4 °C, and the supernatant was collected. Protein concentration was determined using the BCA Protein Assay Kit (Epizyme Biotech, Shanghai, China). For SDS-PAGE, the samples were mixed with 5X SDS-PAGE loading buffer (Beyotime Biotechnology, Shanghai, China) in a 4:1 ratio, heated at 100 °C for 10 min, and subsequently stored at -80 °C.

Proteins were separated by SDS-PAGE and transferred onto PVDF membranes. After blocking at room temperature, the membranes were incubated overnight at 4 °C with primary antibodies against Nrf2 (1:2000, Proteintech, China), HO-1 (1:1000, Proteintech, China), NLRP3 (1:5000, Proteintech, China), and β-actin (1:5000, Proteintech, China). Following incubation with a secondary antibody (rabbit, 1:5000, Proteintech, China) at room temperature for 1 h, the membranes were developed using an ECL kit (Meilun Biotechnology, Dalian, China) in a BIO-OI gel imaging system (Optical Instrument Biotechnology, Guangzhou, China). Immunoreactive bands were visualized and analyzed using ImageJ software.

### Statistical analysis

Data are presented as the mean ± standard deviation (SD), with a sample size of n ≥ 3. Statistical significance was assessed using one-way analysis of variance (ANOVA) and independent t-tests, performed with GraphPad Prism 9.5. Significance levels were indicated as follows: *P < 0.05, ** P < 0.01, *** P < 0.001, **** P < 0.0001, while *ns* denotes *P* > 0.05, indicating no significant difference.

## Results and Discussion

### Preparation and characterization of the Fe-Cur NPs

**Figure 1:**
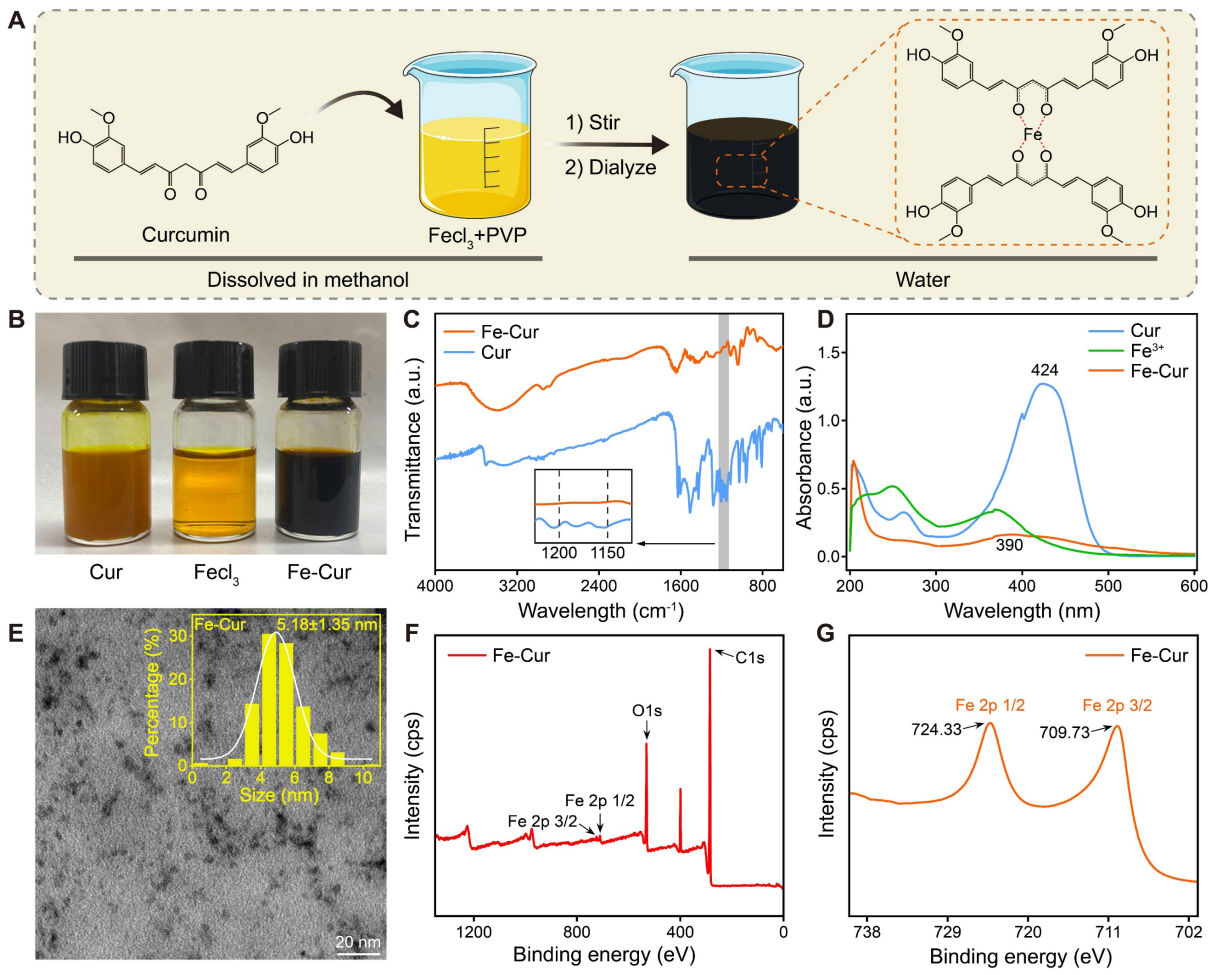
** Preparation and characterization of Fe-Cur NPs. (A)** Schematic diagram of the synthesis of Fe-Cur NPs. **(B)** Photographs of Cur, Fecl_3_, and Fe-Cur NPs dissolved in methanol solution. **(C)** FTIR spectra of Cur and Fe-Cur NPs. **(D)** UV-vis spectra of Cur, Fecl_3_, and Fe-Cur NPs in methanol. **(E)** TEM image of Fe-Cur NPs and particle size statistics chart. **(F)** XPS spectra of all elements in Fe-Cur NPs. **(G)** XPS spectra of Fe in Fe-Cur NPs.

Curcumin has gained significant attention due to its potent anti-inflammatory and antioxidant properties, but its low bioavailability limits its therapeutic applications 25. In this study, we synthesized Fe-Cur NPs that exhibit complete water solubility. By adding iron ions to the curcumin's methanol solution, the solution changed from yellow to black-brown upon stirring, indicating successful coordination between ferric ions and the phenol group of curcumin 26. PVP was incorporated to enhance nanoparticle stability, and dialysis was performed to remove unreacted iron ions, yielding well-dispersed Fe-Cur NPs in an aqueous solution **(Figure 1A-B)**.

The successful synthesis of Fe-Cur NPs was confirmed through various characterization techniques. The FTIR spectra of Fe-Cur NPs showed a significant decrease in the infrared intensity at 1150-1200 cm^-1^ (HO-C stretching band), indicating the coordination between Fe^3+^ and the HO-C moieties of curcumin **(Figure 1C)**. UV-vis spectroscopy revealed an absorption peak of around 390 nm for the Fe-Cur NPs group, confirming the synthesis of Fe-Cur NPs **(Figure 1D)**. TEM images indicated that the nanoparticles had an average diameter of approximately 5.18 ± 1.35 nm **(Figure 1E)**. The XPS spectrum reveals that the Fe-Cur NPs are primarily composed of carbon (C), oxygen (O), and iron (Fe) **(Figure 1F)**. Notably, two strong binding energy peaks for iron, corresponding to Fe 2p_3/2_ and Fe 2p_1/2_, are observed at 710 eV and 724 eV, respectively, indicating that the oxidation state of the iron ions remains unchanged during the synthesis process **(Figure 1G)**. Additionally, Fe-Cur NPs exhibited excellent stability in different buffer solutions (H_2_O, PBS, DMEM) with no significant changes over 5 days **(Figure S1)**. These results demonstrate the successful preparation of stable, water-soluble Fe-Cur NPs, offering a promising solution for enhancing the bioavailability of curcumin.

### Preparation and characterization of GM@Fe-Cur/PDA/MH

**Figure 2:**
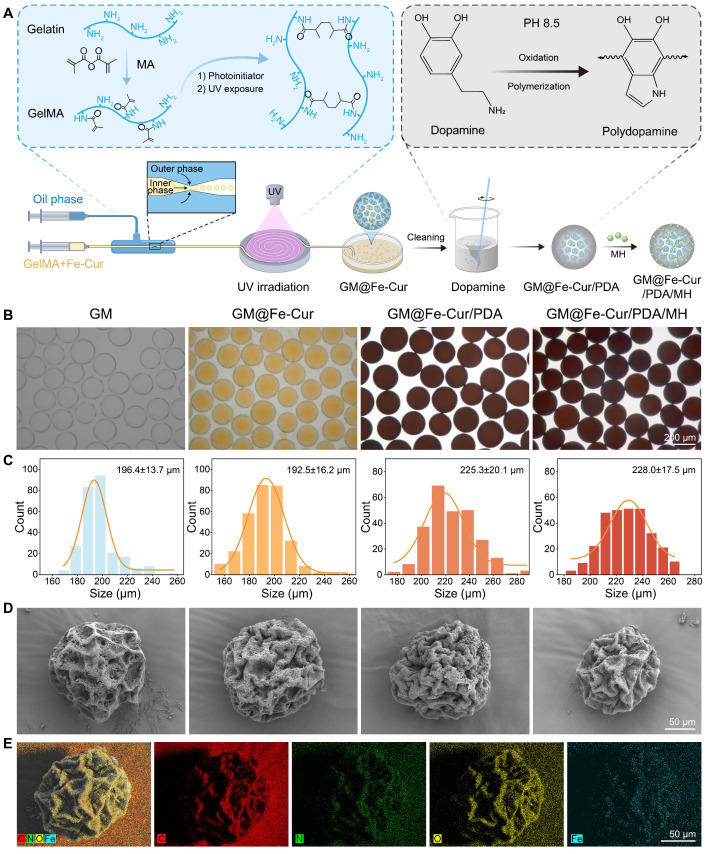
** Preparation and characterization of GM@Fe-Cur/PDA/MH. (A)** Schematic illustration of the synthesis process for GM@Fe-Cur/PDA/MH. **(B)** White light images of the four microspheres. **(C)** Particle size distribution chart for the four types of microspheres. **(D)** SEM images of the four freeze-dried microsphere samples. **(E)** Elemental composition analysis of GM@Fe-Cur/PDA/MH.

GelMA is a gelatin derivative modified with olefin double bonds, which can be rapidly cured into a gel by ultraviolet and visible light in the presence of photoinitiators. Due to its excellent biocompatibility and formability, GelMA has been widely used in biological tissue engineering 27. In this study, microspheres were synthesized using a microfluidic technique with GelMA as the matrix. This technology offers a significant advantage in producing monodisperse microspheres with precise particle sizes, narrower size distribution, and enhanced reproducibility 28. By adjusting the flow rates of the inner phase (a mixed solution of GelMA and Fe-Cur NPs) and the outer phase (an oil phase), monodisperse microspheres with an approximate diameter of 200 μm were prepared **(Figure 2A).**

The GM were initially colorless and transparent. Upon incorporating Fe-Cur NPs, the microspheres exhibited a yellow coloration, indicating uniform dispersion of Fe-Cur NPs within the microspheres and without any significant change in their mean diameter **(Figure 2B).** To enhance the adhesion ability of the microspheres in periodontal tissues, the surface of the microspheres was modified with PDA, a biocompatible substance inspired by mussel adhesion properties 29, 30. This modification resulted in microspheres with an overall black color and a slightly enlarged diameter of approximately 225 μm. The PDA-modified material exhibited many active sites on its surface, which could be utilized for further functionalization by grafting thiol-, amino-, and hydroxyl-containing substances *via* covalent or non-covalent interactions 31. MH was selected to expand the antibacterial functionality of the composite microspheres. MH interacts electrostatically with PDA and adsorbs on the surface of the microspheres through π-π interaction, imparting antimicrobial properties to the microspheres 32. The microspheres' diameter remained unchanged following MH loading **(Figure 2C)**. SEM revealed that all four types of microspheres exhibited a wrinkled surface morphology after freeze-drying. Elemental distribution maps of GM@Fe-Cur/PDA/MH, generated using EDS, demonstrated the uniform distribution of C, N, O, and Fe within the microspheres **(Figure 2D-E)**. These findings confirmed the successful preparation of GM@Fe-Cur/PDA/MH microspheres **(Figure S2)**.

### Adhesion, injectability, and degradation behavior of GM@Fe-Cur/PDA/MH

The oral cavity is a highly dynamic environment, presenting significant challenges for the localized administration of drugs in treating periodontitis. However, numerous studies inspired by the adhesive properties of mussels have shown promising results 33. In this study, the surface of the microspheres was modified using PDA, and the adhesion ability of GM@Fe-Cur/PDA/MH was evaluated through various methods.

As shown in **Figure S3A**, GM@Fe-Cur/PDA/MH demonstrated strong adhesion to various surfaces, including human-isolated teeth, metals, glass, and plastic. Moreover, the microspheres adhered well to rat' isolated maxillary gingival tissue, even after overturning and twisting **(Figure S3B)**. In patients with periodontitis, inflammation significantly increases the amount of gingival crevicular fluid. While this fluid aids in local defense by assisting in cleaning, it can also weaken the retention of drugs at the periodontitis site, leading to suboptimal treatment outcomes 34. PDA has exceptional underwater adhesion capabilities, forming a robust adhesive layer with other surfaces through hydrogen bonding, ionic bonding, and hydrophobic interactions 31. Human-isolated teeth were used to simulate the natural oral environment and further evaluate the adhesion properties of GM@Fe-Cur/PDA/MH. The microspheres were tested under different water flow rates, with saliva flow rates used as a proxy due to limited data on gingival crevicular fluid flow rates 35. The study found that the microspheres demonstrated a retention rate of approximately 70.6% to 62.5% within 10 min under flow rates of 0.3 mL/min and 0.6 mL/min **(Figure S3C)**. Even at a 0.9 mL/min flow rate, within the range of stimulated saliva flow, the retention rate remained at nearly 50.6% **(Figure S3D)**. These results suggest that GM@Fe-Cur/PDA/MH can withstand the cleansing action of gingival sulcus fluid, indicating a promising potential for periodontitis treatment. In addition, the injectability of GM@Fe-Cur/PDA/MH microspheres was also tested. The microspheres could pass a standard small-volume white pipette tip smoothly without noticeable clogging. During injection, the size and morphology of the microspheres were visible, ultimately forming ideal droplets **(Figure S4)**, demonstrating their good operational handling and injectability.

Degradation is a critical factor influencing the practical application of biomaterials. This study immersed microspheres in PBS, artificial saliva, or artificial saliva containing 100 ng/mL LPS to evaluate their degradation behavior. As shown in **Figure S5A**, PBS, as a conventional buffer solution, had little effect on the degradation of the GelMA matrix. The microspheres exhibited slow changes in morphology and structure, gradually fading color. In artificial saliva, the microspheres maintained their morphology and structural stability, with a gradual lightening of color, blurring of the surface, and slight degradation **(Figure S5B)**. Artificial saliva primarily consists of salivary amylase and inorganic salts, with amylase targeting carbohydrates rather than GelMA, a modified collagen derivative. Therefore, the partial degradation observed may be attributed to ions or trace components in the artificial saliva.

Upon the addition of LPS, the degradation rate of the microspheres increased. The surface morphology became irregular, and mild degradation was observed in localized areas, though the overall structure remained intact **(Figure S5C)**. As a bacterial endotoxin, LPS triggers strong immune responses. *In vivo,* LPS can activate localized inflammatory responses, enhance enzymatic activity, or induce the secretion of degradative enzymes, thereby promoting GelMA degradation. Although this study did not involve immune cells, LPS may also alter the surface properties of PDA, contributing to degradation to a certain extent. In summary, GM@Fe-Cur/PDA/MH exhibited excellent adhesion, injectability, and controlled degradation, highlighting its promising potential for application in periodontitis treatment.

### *In vitro* drug release behavior of GM@Fe-Cur/PDA/MH

The LC and EE of MH in GM@Fe-Cur/PDA/MH were determined by UV-vis absorbance measurements. The LC of MH was found to be 1.21% ± 0.07%, and the EE was 53.04% ± 3.36%, indicating that MH was successfully and efficiently loaded onto the surface of the microspheres. The LR of Fe-Cur NPs was 82.15 ± 2.16%, demonstrating that most Fe-Cur NPs were effectively incorporated into the microsphere matrix. To evaluate the therapeutic potential of GM@Fe-Cur/PDA/MH, the cumulative release behaviors of MH and Fe-Cur NPs were measured in three different environmental media **(Figure S6)**.

PBS, as a stable medium, simulates non-inflammatory physiological conditions. Microsphere degradation primarily occurs through the hydrolysis and swelling of the GelMA matrix. The release of Fe-Cur NPs within the microspheres is controlled by slow diffusion, resulting in a gradual release rate. After 96 h, the cumulative release of Fe-Cur NPs reached 3.92%. MH, located on the outer layer of the microspheres, exhibited a faster release profile, with a cumulative release of 42.47%.

In artificial saliva containing various compounds and enzymes, the release rates of MH and Fe-Cur NPs were accelerated. These components may alter the pH of the microenvironment or affect the stability of PDA with MH or GelMA with PDA, thereby enhancing the release. After 96 h, the cumulative release of MH increased to 57.80%, while the release of Fe-Cur NPs reached 6.96%. According to the degradation results, adding LPS slightly enhanced the surface degradation of the microspheres. Consequently, in the LPS environment, the cumulative release of MH further increased to 60.08%, while the release of Fe-Cur NPs showed a slight increase to 7.28%.

These findings indicate that the GM@Fe-Cur/PDA/MH microspheres exhibit stable and sustained drug release behavior under normal physiological conditions. When environmental conditions change, such as in artificial saliva or LPS-containing environments, the drug release rates accelerate, particularly for MH. This demonstrates the microspheres' ability to respond to environmental changes, showcasing their controllable release properties. Such characteristics highlight the potential of GM@Fe-Cur/PDA/MH microspheres for targeted treatment of periodontitis.

### Biocompatibility of GM@Fe-Cur/PDA/MH

hPDLSCs are mesenchymal stem cells (MSCs) derived from the periodontal ligament, exhibiting self-renewal and multipotent differentiation capabilities. These cells have shown potential for promoting new bone formation in periodontal tissues 36. In **Figure S7A**, the periodontal ligament tissue appears yellowish-brown, with spindle-shaped cells exhibiting the typical morphology of MSCs. Flow cytometry analysis revealed high expression levels of MSC surface markers, CD90 (99.92%) and CD105 (99.74%). while the markers CD45 (0.22%) and CD31 (0.31%), associated with hematopoietic stem cells and other non-MSC types, were significantly under-expressed 37 **(Figure S7B)**. These results confirm that the isolated and cultured hPDLSCs possess the characteristics and potential for osteogenic differentiation, supporting their suitability for bone regeneration and periodontal therapy applications.

**Figure 3:**
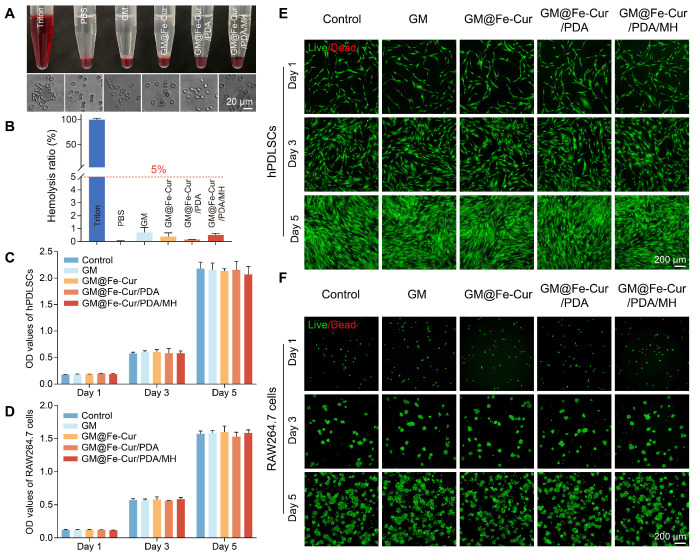
***In vitro* hemocompatibility and cytocompatibility of microspheres. (A)** Photographs of microspheres after incubation with blood and morphology of the corresponding erythrocytes. **(B)** Hemolysis rate of microspheres. CCK-8 assay of microspheres at 1, 3, and 5 days in **(C)** hPDLSCs and **(D)** RAW264.7 cells. Fluorescent images of live/dead cell staining in **(E)** hPDLSCs and **(F)** RAW264.7 cells for 1, 3, and 5 days.

The biocompatibility of biomaterials is a crucial consideration in their design and development, as it directly influences their safe and effective application within the body. The gingiva is susceptible to mechanical stimulation and bleeding in inflammatory conditions due to capillary dilation and increased permeability 38. Therefore, fresh rabbit blood was used to simulate the interaction between microspheres and blood to test the blood compatibility of microspheres. As 0.1% TritonX-100 is commonly used for cell lysis, red blood cells in the Triton group were fully lysed, setting the hemolysis rate at 100%. In contrast, no significant hemolysis was observed in any of the microsphere groups (GM, GM@Fe-Cur, GM@Fe-Cur/PDA, GM@Fe-Cur/PDA/MH), with hemolysis rates below the safety threshold of 5%. No significant difference was observed between these and the PBS groups, indicating good blood compatibility **(Figure 3A-B)**.

Furthermore, the cytocompatibility of the microspheres was also evaluated using hPDLSCs and RAW264.7 cells. The CCK-8 assay, conducted on the 1st, 3rd, and 5th days of culture, demonstrated no significant difference in cell viability between the microsphere-treated and the control groups for both cell types **(Figure 3C-D)**. Live/dead cell staining experiments revealed an increase in cell density with incubation time, while the number of red dead cells did not significantly rise, and cell morphology remained unchanged **(Figure 3E-F)**. These results indicate that the microspheres did not adversely affect the viability of various cell types and demonstrated excellent cytocompatibility. In summary, the findings confirm that GM@Fe-Cur/PDA/MH microspheres possess superior blood and cytocompatibility, supporting their suitability for safe and effective use in the periodontal environment.

### *In vitro* antioxidant properties of GM@Fe-Cur/PDA/MH

**Figure 4:**
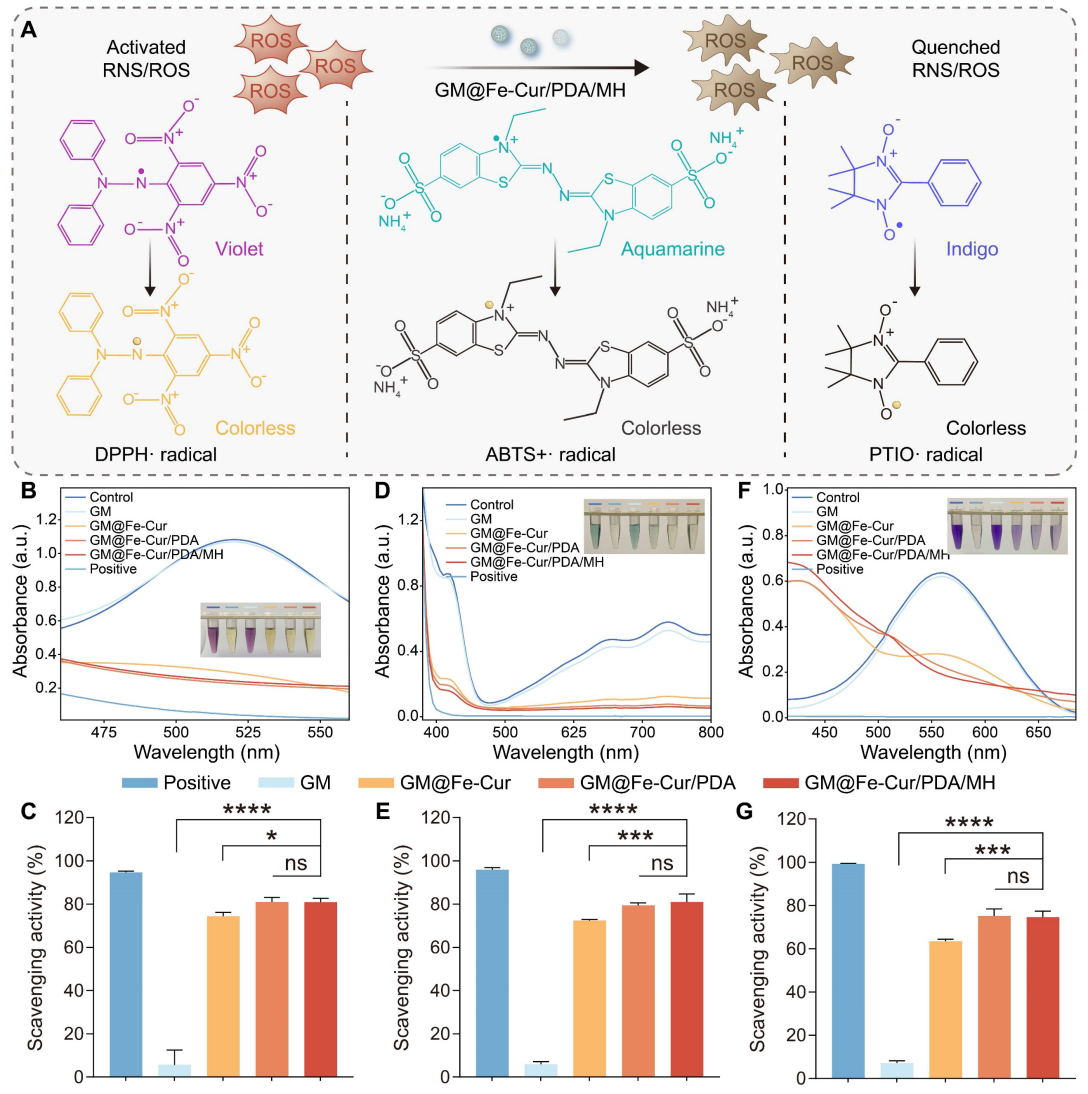
***In vitro* free radical scavenging ability of microspheres. (A)** Schematic representation of the scavenging process for three types of free radicals (DPPH·, ABTS+·, and PTIO·). **(B)** UV-vis spectra of the DPPH· radical after incubation with microspheres. **(C)** Quantification of the DPPH· radical scavenging rate. **(D)** UV-vis spectra of the ABTS+· radical after incubation with microspheres. **(E)** Quantification of the ABTS+· radical scavenging rate. **(F)** UV-vis spectra of the PTIO· radical after incubation with microspheres. **(G)** Quantification of the PTIO· radical scavenging rate.

Curcumin, a natural reducing agent extracted from the rhizome of turmeric, contains various functional groups, including phenolic hydroxyl and methoxy groups 39. These functional groups confer upon curcumin a powerful free radical scavenging ability and the capacity to inhibit oxidative stress. The anti-oxidative ability of GM@Fe-Cur/PDA/MH was tested *via* various probes, including 1,1-diphenyl-2-trinitrophenylhydrazine radical (DPPH·), 2,2′-azobis (3-ethylbenzothiazoline-6-sulfonic acid) radical (ABTS+·), and 2-Phenyl-4,4,5,5-tetramethylimidazoline-1-oxyl-3-oxide radical (PTIO·) **(Figure 4A)**.

DPPH·, a stable nitrogen-centered free radical, is frequently employed to assess the antioxidant capacity of substances. Its alcoholic solution is purple, with strong absorption at 515 nm. In the presence of antioxidants, the solution's color fades, leading to a decrease in absorbance at 515 nm. Upon adding microsphere extracts, the Fe-Cur NPs loaded microsphere group exhibited significant free radical scavenging activity, reaching approximately 74%. Notably, the GM@Fe-Cur/PDA and GM@Fe-Cur/PDA/MH groups demonstrated higher scavenging effects, exceeding 80% **(Figure 4B-C)**. This result suggests that the PDA layer, with its reducing functional groups, also contributed to the scavenging activity. Similarly, the ABTS+· radical, another nitrogen-centered radical, is widely used in indirect antioxidant detection methods. Microsphere extracts reacting with ABTS+· result in a decrease in absorbance at 405 nm. The results revealed that GM@Fe-Cur/PDA/MH displayed excellent nitrogen-centered free radical scavenging effects **(Figure 4D-E)**.

Furthermore, the PTIO· radical, an oxygen-centered radical known for its stability, was used to assess the extent of ROS scavenging by the antioxidants. As expected, significant color fading was observed in the GM@Fe-Cur, GM@Fe-Cur/PDA, and GM@Fe-Cur/PDA/MH groups **(Figure 4F-G)**, indicating that these composite microspheres possess outstanding ROS scavenging activity. These findings confirm that GM@Fe-Cur/PDA/MH functions as a potent free radical scavenger, highlighting its potential as a promising candidate for anti-inflammatory treatment in periodontitis.

### Ability to scavenge intracellular ROS

Previous experiments have demonstrated that GM@Fe-Cur/PDA/MH exhibits excellent antioxidant capacity; thus, the scavenging behavior of these microspheres on ROS within cells was further investigated. The oxidative stress microenvironment can cause severe damage to periodontal tissues and is a significant barrier to periodontal therapy 2. Consequently, a hydrogen peroxide solution was used to simulate an oxidative stress environment 40. DCFH-DA detected the impact of GM@Fe-Cur/PDA/MH on intracellular ROS levels. Furthermore, the protective efficacy of GM@Fe-Cur/PDA/MH against oxidative stress-induced cell death was evaluated through live/dead cell staining and the CCK-8 assay.

**Figure 5:**
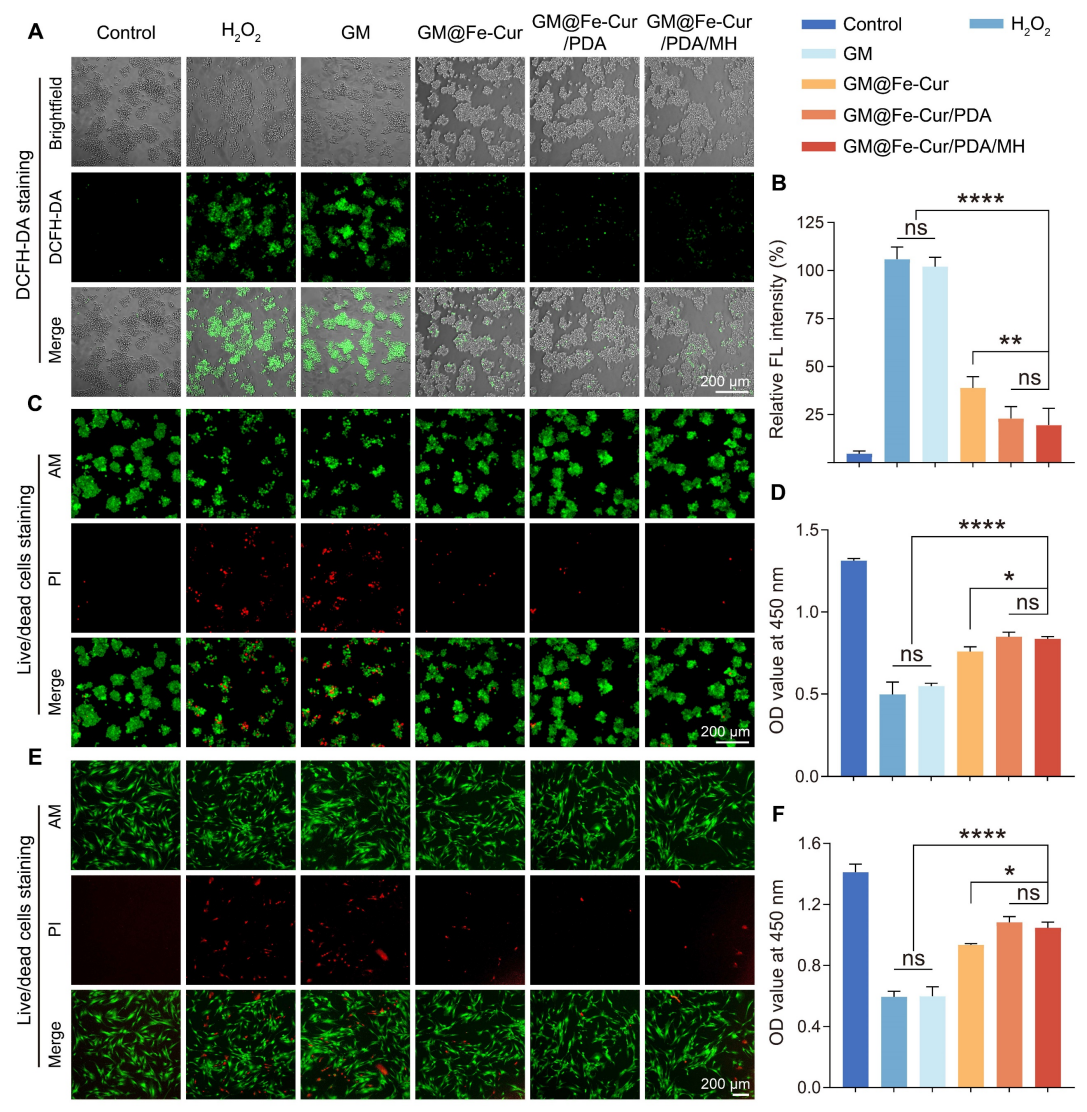
** Effect of microspheres on scavenging intracellular ROS under H_2_O_2_-induced oxidative stress. (A)** DCFH-DA-stained fluorescent images of RAW264.7 cells after treatment with different microspheres. **(B)** Quantitative analysis of DCFH-DA fluorescence intensity. **(C)** Live/dead cell staining and **(D)** CCK-8 assay of RAW264.7 cells under oxidative stress conditions. **(E)** Live/dead cell staining and **(F)** CCK-8 assay of hPDLSCs under oxidative stress conditions.

Following the incubation of RAW264.7 cells with 100 mM H_2_O_2_, a substantial amount of intracellular ROS was produced, as evidenced by the bright DCF fluorescence. In contrast, only minimal fluorescence was observed in the GM@Fe-Cur, GM@Fe-Cur/PDA, and GM@Fe-Cur/PDA/MH groups, indicating that the majority of intracellular ROS were effectively cleared **(Figure 5A-B)**. Subsequent live/dead cell staining revealed a significant number of dead cells (red fluorescence) in the H_2_O_2_ group and the GM group. In contrast, GM@Fe-Cur, GM@Fe-Cur/PDA, and GM@Fe-Cur/PDA/MH groups exhibited markedly fewer dead cells, demonstrating significant protection against oxidative stress-induced cell death, as confirmed by the CCK-8 assay results **(Figure 5C-D)**. The hPDLSCs showed similar outcomes when exposed to H_2_O_2_-induced oxidative stress. In the H_2_O_2_ and GM groups, significant cell death was observed, with surviving cells displaying a shriveled morphology, in contrast to the spindle-like shape characteristic of the control group **(Figure 5E)**. However, after treatment with microsphere extracts, there was a notable reduction in cell death, accompanied by partial restoration of normal cell morphology **(Figure 5F)**. These findings suggest that GM@Fe-Cur/PDA/MH effectively scavenges intracellular ROS and mitigates the cellular damage caused by oxidative stress.

### NIR-photothermal properties of GM@Fe-Cur/PDA/MH

**Figure 6:**
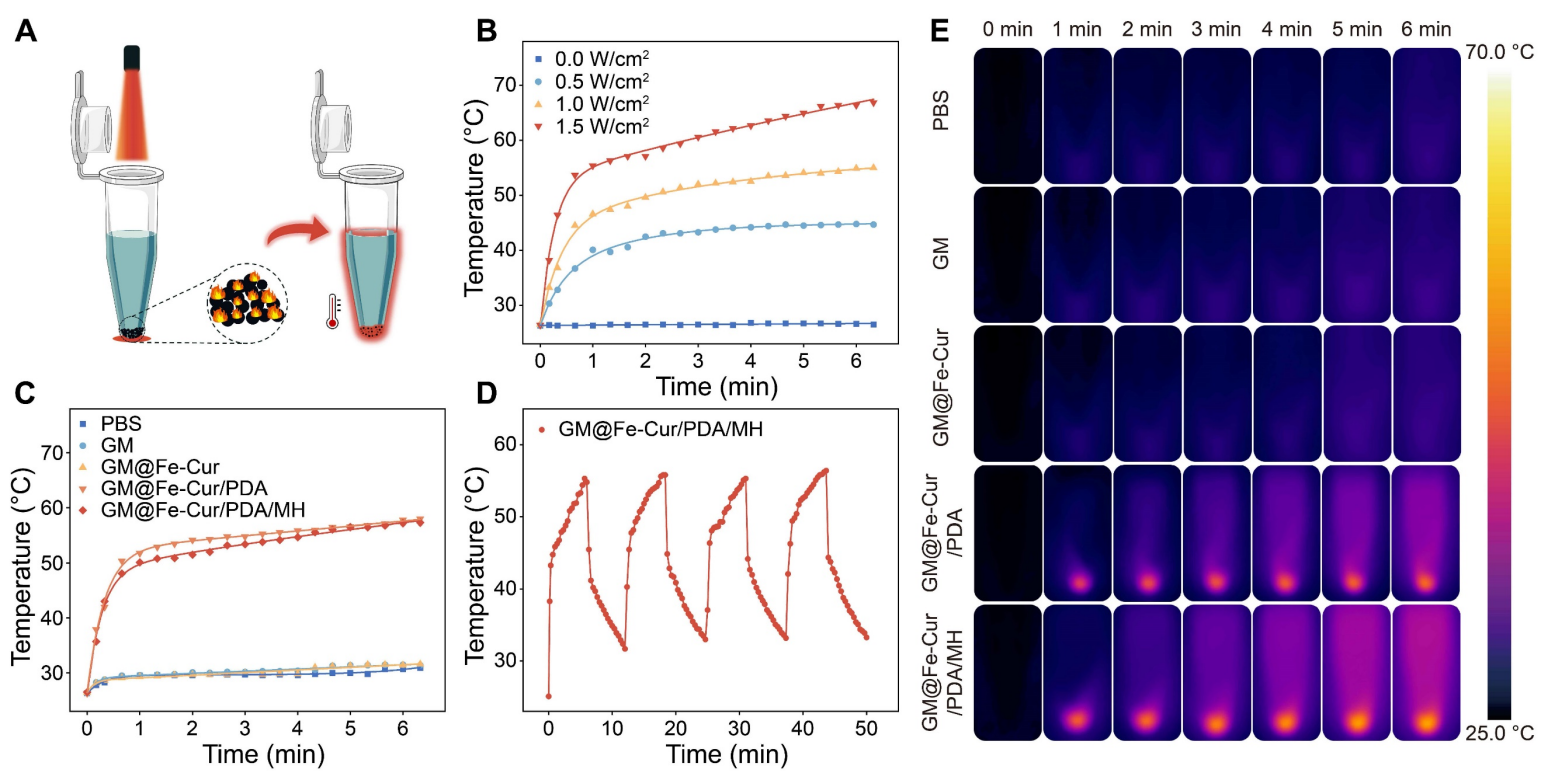
** NIR photothermal performance of GM@Fe-Cur/PDA/MH. (A)** Schematic illustration of photothermal therapy. **(B)** Temperature variation curves of GM@Fe-Cur/PDA/MH at different powers. **(C)** Temperature variation curves of PBS, GM, GM@Fe-Cur, GM@Fe-Cur/PDA, and GM@Fe-Cur/PDA/MH under 1 W/cm^2^ NIR laser irradiation. **(D)** Photothermal stability of GM@Fe-Cur/PDA/MH after four cycles of 1 W/cm^2^ NIR laser irradiation. **(E)** Infrared thermography images of different microspheres during NIR laser irradiation at 1 W/cm^2^.

By absorbing NIR light, photothermal materials convert light energy into heat 41. PDA, a notable photothermal material, exhibits high NIR absorbance and exceptional photothermal conversion efficiency 42. Here, microspheres were subjected to NIR (808 nm) irradiation for approximately 6 min **(Figure 6A)**. The temperature of the GM@Fe-Cur/PDA/MH group increased with increasing NIR optical power **(Figure 6B)**. At an intensity of 1 W/cm^2^, the temperature of GM@Fe-Cur/PDA/MH rose from 26.5 °C to 55 °C within approximately 6 min, surpassing the sterilizing temperature (≥ 50 °C) while remaining within the oral cavity's high-temperature tolerance range (55-60 °C), which does not cause harm to periodontal tissues 43. At 0.5 W/cm^2^, the microspheres reached a maximum temperature of 45 °C, which may be insufficient for effective bacterial eradication. Conversely, at 1.5 W/cm^2^, the temperature rapidly escalated to approximately 60 °C within 2 min, eventually reaching around 67 °C, which could induce a heat stress response and damage healthy tissues. Based on these findings, further tests were conducted on other microsphere groups and the PBS group at a laser intensity of 1 W/cm^2^ **(Figure 6C and Figure 6E)**. The results demonstrated that the PBS, GM, and GM@Fe-Cur groups exhibited the least temperature fluctuations, while the temperature profile of the GM@Fe-Cur/PDA group was no different from that of the GM@Fe-Cur/PDA/MH group. This suggests that the photothermal conversion efficacy of the microspheres was achieved through the PDA and that the incorporation of additional substances did not significantly alter or affect the photothermal performance. Finally, after four on/off light cycles, the GM@Fe-Cur/PDA/MH group's temperature change curves remained stable **(Figure 6D)**. These results demonstrate that GM@Fe-Cur/PDA/MH exhibits stable and superior photothermal performance.

In summary, the photothermal conversion induced by NIR light *via* PDA in the GM@Fe-Cur/PDA/MH microspheres effectively generates heat capable of achieving sterilizing temperatures without harming periodontal tissues. This provides a promising approach for antibacterial therapy. The stable photothermal properties further underscore the potential of GM@Fe-Cur/PDA/MH microspheres in therapeutic applications.

### *In vitro* antibacterial properties

**Figure 7:**
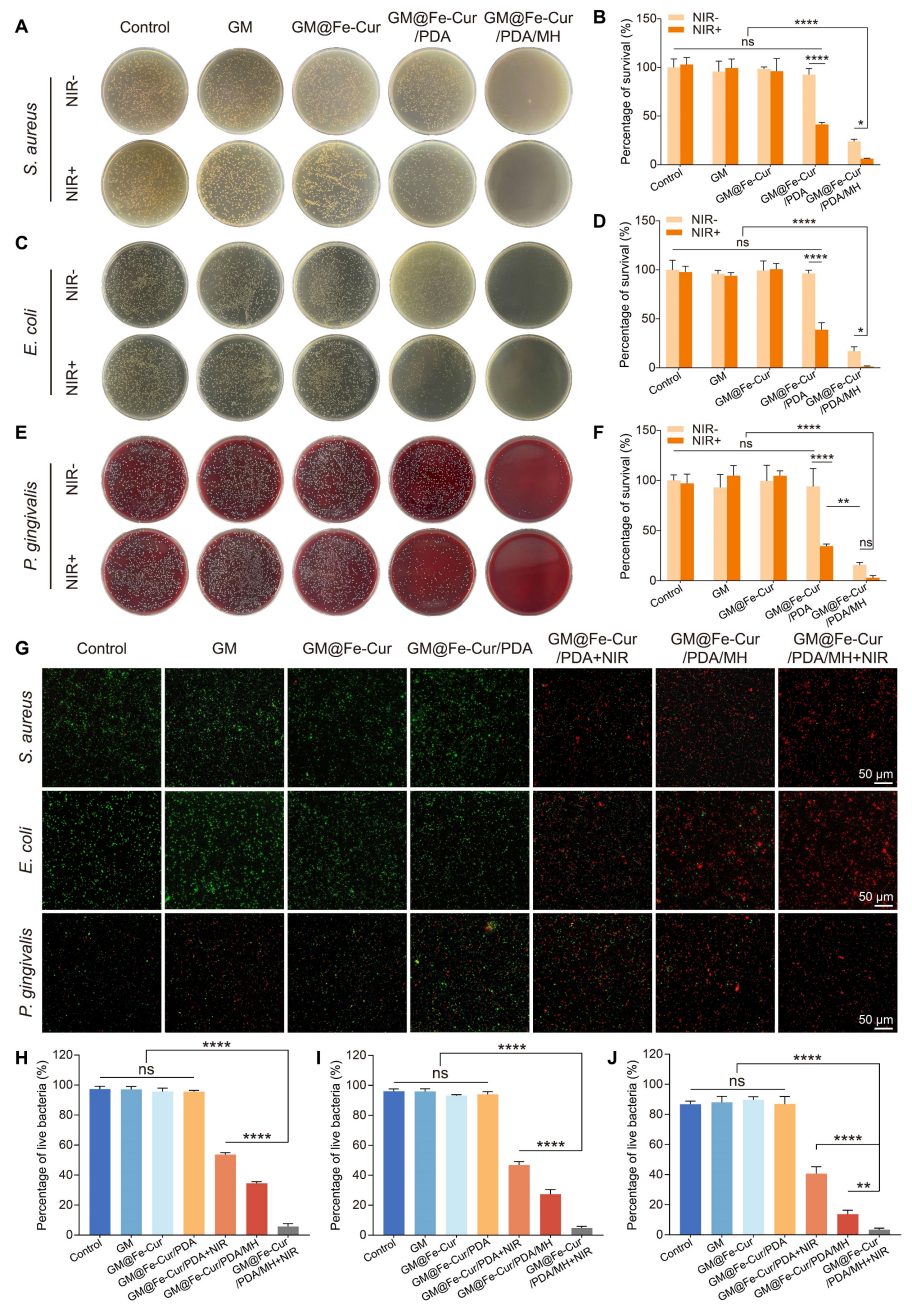
** Antimicrobial properties of microspheres *in vitro*.** Photographs showing colony-forming units of **(A)** *S. aureus*, **(C)** *E. coli*, and **(E)** *P. gingivalis* following different microsphere treatments. Antibacterial activity against **(B)** *S. aureus*, **(D)** *E. coli*, and **(F)** *P. gingivalis*. **(G)** Live/dead staining images of *S. aureus*, *E. coli*, and *P. gingivalis* after different microsphere treatments. Quantitative analysis of fluorescent images for **(H)** *S. aureus*, **(I)** *E. coli*, and **(J)** *P. gingivalis*.

Periodontitis is an infectious disease caused by a variety of pathogenic bacteria. These bacteria form a complex community structure with each other and possess a certain resistance to antimicrobial drugs, which presents a significant challenge in the antimicrobial treatment of periodontitis 44. To evaluate the antimicrobial effects induced by microspheres and NIR irradiation, two model bacteria, *S. aureus,* and *E. coli*, were selected and co-cultured with the microspheres. As shown in **Figure 7A and Figure 7C**, the GM@Fe-Cur/PDA/MH group demonstrated robust antibacterial activity against both *S. aureus* and *E. coli*, attributed to the presence of MH. However, a certain number of bacteria still survived. Following NIR irradiation, the colonies were reduced to 6.4% and 1.4%, respectively, indicating almost complete bacterial eradication. In contrast, the survival rates of *S. aureus* and *E. coli* in the GM@Fe-Cur/PDA group were 42.5% and 39%, respectively, post-NIR irradiation **(Figure 7B and Figure 7D)**. These findings underscore that while NIR-induced photothermal conversion has a significant antibacterial effect, it alone is insufficient for complete bacterial eradication. Similarly, antimicrobial testing of microspheres was performed against *P. gingivalis*, the primary pathogenic bacterium of periodontal diseases **(Figure 7E-F)**. The results showed no significant change in bacterial viability in the microsphere group without photothermal effect or MH loading. However, when subjected to NIR irradiation or MH loading, it still showed an excellent antibacterial effect. The combination of NIR irradiation and MH drastically reduced the bacterial survival rate, which can effectively eradicate *P. gingivalis.*

SYTO-9/PI staining was used to investigate the antibacterial properties of GM@Fe-Cur/PDA/MH **(Figure 7G)**. In the control, GM, GM@Fe-Cur, and GM@Fe-Cur/PDA groups (without NIR), green fluorescence predominated, with minimal red fluorescence, indicating low bacterial death. However, in the GM@Fe-Cur/PDA group, dead bacteria significantly increased post-NIR irradiation. Conversely, the GM@Fe-Cur/PDA/MH group (with NIR) exhibited predominantly red and significantly reduced green fluorescence, indicating near-total bacterial death and the most effective antimicrobial effect **(Figure 7H-J)**.

In conclusion, neither antibiotics nor NIR treatment alone can completely eradicate bacteria, but their combined use produces a significant synergistic antibacterial effect. With its ability to penetrate tissues, NIR effectively overcomes the challenge posed by biofilm barriers that prevent antibiotics from reaching and killing deep-seated bacteria while offering a potential solution to antibiotic resistance. These findings demonstrate that GM@Fe-Cur/PDA/MH exhibits potent antibacterial activity against various bacterial strains, highlighting its significant potential for application in the treatment of periodontitis.

### The immunomodulatory activity of GM@Fe-Cur/PDA/MH on RAW246.7 cells

**Figure 8:**
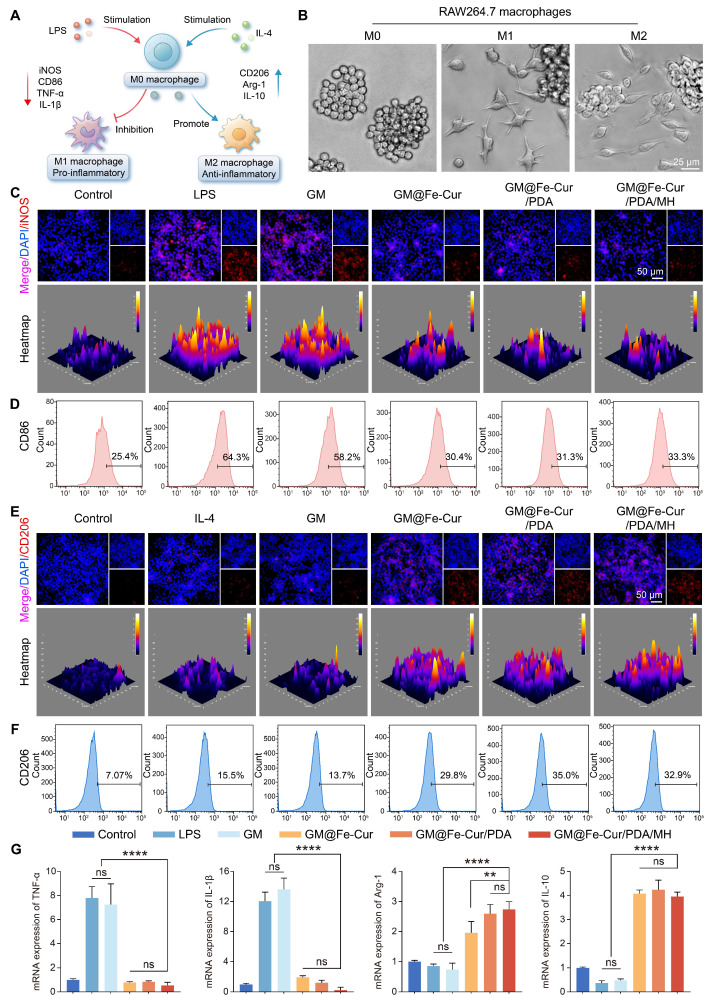
** Regulation of macrophage polarization by GM@Fe-Cur/PDA/MH. (A)** Schematic diagram of GM@Fe-Cur/PDA/MH inhibiting M1 macrophage polarization while promoting M2 polarization. **(B)** Morphological changes in RAW264.7 cells under different polarization conditions. Immunofluorescence images of **(C)** M1 (iNOS) and **(E)** M2 (CD206) macrophage markers. Flow cytometry analysis of macrophage polarization markers: **(D)** CD86 for M1 macrophages and **(F)** CD206 for M2 macrophages. **(G)** mRNA expression levels of pro-inflammatory and anti-inflammatory cytokines *in vitro*.

Macrophages can polarize into distinct functional states in response to environmental signals, primarily into M1 (pro-inflammatory) and M2 (anti-inflammatory) phenotypes 8. Upon tissue injury, macrophages transition to the M1 type, secreting pro-inflammatory cytokines and chemokines that trigger a robust inflammatory response. Once the damage is under control, macrophages transition to the M2 type, secreting growth factors and anti-inflammatory mediators to suppress inflammation and initiate tissue repair 45. To investigate the regulatory role of GM@Fe-Cur/PDA/MH on inflammatory responses, phenotypic changes in macrophages were assessed *via* immunofluorescence and flow cytometry. Under stimulation with LPS and IL-4, macrophages exhibited notable morphological changes **(Figure 8B)**. LPS-induced M1 macrophages displayed prominent irregular pseudopodia, while IL-4-induced M2 macrophages had smooth edges and a spindle-like shape.

In immunofluorescence staining, inducible nitric oxide synthase (iNOS) and the mannose receptor (CD206) were used as markers for M1 and M2 macrophages, respectively 46 **(Figure 8C and Figure 8E)**. The LPS group showed a marked increase in iNOS fluorescence, whereas the IL-4 group displayed heightened CD206 fluorescence. Treatment with GM@Fe-Cur, GM@Fe-Cur/PDA, and GM@Fe-Cur/PDA/MH significantly reduced iNOS fluorescence intensity, while CD206 fluorescence was substantially enhanced. These findings were further corroborated by flow cytometric analysis of the M1-specific marker CD86 and the M2-specific marker CD206 **(Figure 8D and Figure 8F)**. In the GM@Fe-Cur/PDA/MH treatment group, the proportion of CD86-positive cells decreased from 64.3% in the LPS group to 33.3%, demonstrating a strong inhibitory effect. Concurrently, the percentage of CD206-positive cells increased from 7.07% in the control group to 32.9%, indicating a pronounced promotion of M2 polarization by GM@Fe-Cur/PDA/MH. Thus, following treatment with GM@Fe-Cur/PDA/MH, the proportion of irregular M1-type cells significantly decreased, while the percentage of spindle-shaped M2-type cells markedly increased.

Given the critical role of inflammatory cytokine expression in macrophage polarization, qRT-PCR was used to evaluate the regulatory effect of GM@Fe-Cur/PDA/MH on inflammatory mediators, including pro-inflammatory factors TNF-α and IL-1β, and anti-inflammatory factors Arg-1 and IL-10 47 **(Figure 8G)**. Compared to the control group, the LPS group exhibited a significant upregulation of TNF-α and IL-1β, reflecting the potent pro-inflammatory impact of LPS. In contrast, treatment with GM@Fe-Cur, GM@Fe-Cur/PDA, and GM@Fe-Cur/PDA/MH significantly downregulates these pro-inflammatory factors. Additionally, LPS stimulation appeared to suppress the expression of Arg-1 and IL-10, while GM@Fe-Cur/PDA/MH treatment markedly enhanced the expression of these anti-inflammatory mediators.

In summary, GM@Fe-Cur/PDA/MH demonstrated potent immunomodulatory capabilities by significantly inhibiting M1 polarization, promoting M2 macrophage polarization, and regulating inflammation-related cytokine secretion. This immunomodulatory effect is crucial in treating periodontitis, as it helps suppress excessive inflammatory responses and promotes tissue repair and regeneration, offering a potentially innovative strategy for periodontitis therapy.

### Effect of GM@Fe-Cur/PDA/MH on osteogenic differentiation of hPDLSCs under oxidative stress conditions

**Figure 9:**
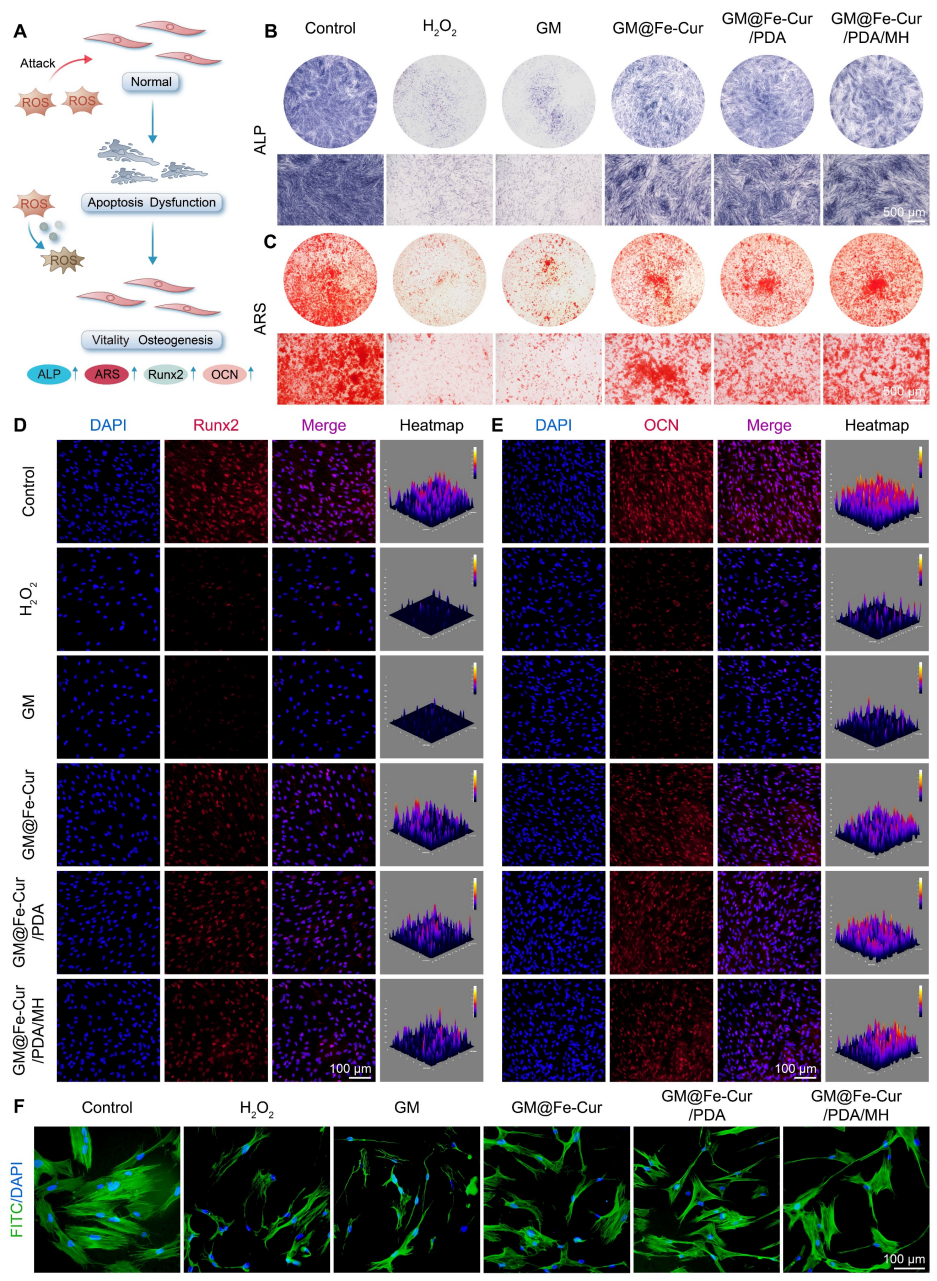
** Effect of GM@Fe-Cur/PDA/MH on the osteogenic differentiation potential of hPDLSCs *in vitro* under oxidative stress conditions. (A)** Schematic representation of GM@Fe-Cur/PDA/MH for the protection of hPDLSCs under oxidative stress conditions. **(B)** ALP-stained images of hPDLSCs on day 7. **(C)** ARS-stained images of hPDLSCs on day 14. **(D)** Immunofluorescence images of Runx2 on day 7 and **(E)** OCN on day 14. **(F)** Cell morphology of hPDLSCs *via* F-actin staining by phalloidin.

hPDLSCs possess multidirectional differentiation potential and are key cells in periodontal tissue repair. However, under inflammatory conditions, persistent oxidative stress may inhibit their osteogenic differentiation, hindering the repair of bone defects 48. ALP staining, which reflects early osteoblast activity, shows the cells starting to express enzymes related to bone formation, while ARS staining marks the maturation stage of osteoblasts, highlighting mineralized bone matrix 49. Using these two methods, the effect of GM@Fe-Cur/PDA/MH on the osteogenic function of hPDLSCs under oxidative stress was evaluated. As illustrated in **Figure 9B**, ALP activity in hPDLSCs exposed to H_2_O_2_ was significantly reduced, with sparse blue staining, indicating suppressed osteogenic activity. However, ALP levels were significantly elevated in the GM@Fe-Cur, GM@Fe-Cur/PDA, and GM@Fe-Cur/PDA/MH groups, indicating restoration of osteogenic activity. Similarly, ARS staining demonstrated that red calcium nodules were markedly reduced in the H_2_O_2_ group, indicating severe inhibition of mineralized bone matrix synthesis. The GM@Fe-Cur, GM@Fe-Cur/PDA, and GM@Fe-Cur/PDA/MH groups rescued this process, forming a considerable number of calcium nodules **(Figure 9C),** suggesting that GM@Fe-Cur/PDA/MH significantly promotes bone matrix mineralization under oxidative stress.

Runt-related transcription factor 2 (Runx2) and osteocalcin (OCN) are critical osteogenic differentiation markers. Runx2 plays a pivotal role in early osteoblast differentiation, promoting the transition of precursor cells to mature osteoblasts. OCN, by binding calcium ions, aids in the deposition of calcium salts in the bone matrix 50. Immunofluorescence staining revealed that treatment with GM@Fe-Cur, GM@Fe-Cur/PDA, and GM@Fe-Cur/PDA/MH alleviated the oxidative stress induced by H_2_O_2_, significantly enhancing the red fluorescence signals of Runx2 and OCN, indicating restored expression of both early and late-stage osteogenic proteins **(Figure 9D-E)**, consistent with the ALP and ARS results. In contrast, the H_2_O_2_ group exhibited markedly weakened fluorescence signals, further confirming the inhibitory effect of oxidative stress on osteogenic differentiation.

The morphology of cells is closely related to their viability, especially for stem cells 51. Under oxidative stress, the morphology of hPDLSCs changed significantly. As shown in **Figure 9F**, the hPDLSCs in the control group exhibited typical spindle shapes, with evenly distributed and intact cytoskeletons and centrally located nuclei. However, after H_2_O_2_ treatment, the cells showed marked shrinkage, disrupted cytoskeletons, and irregular nuclei, indicating significant cellular damage. After treatment with GM@Fe-Cur/PDA/MH, cell morphology improved significantly, with restored cytoskeletal extension, suggesting partial repair of H_2_O_2_-induced damage and a return to near-normal cell morphology. This ability to mitigate oxidative damage and restore osteogenic differentiation under oxidative stress highlights the potential of GM@Fe-Cur/PDA/MH in promoting periodontal tissue mineralization and repair. Thus, these findings provide important therapeutic implications for the treatment of periodontitis.

### GM@Fe-Cur/PDA/MH *in vivo* ROS scavenging capacity

**Figure 10:**
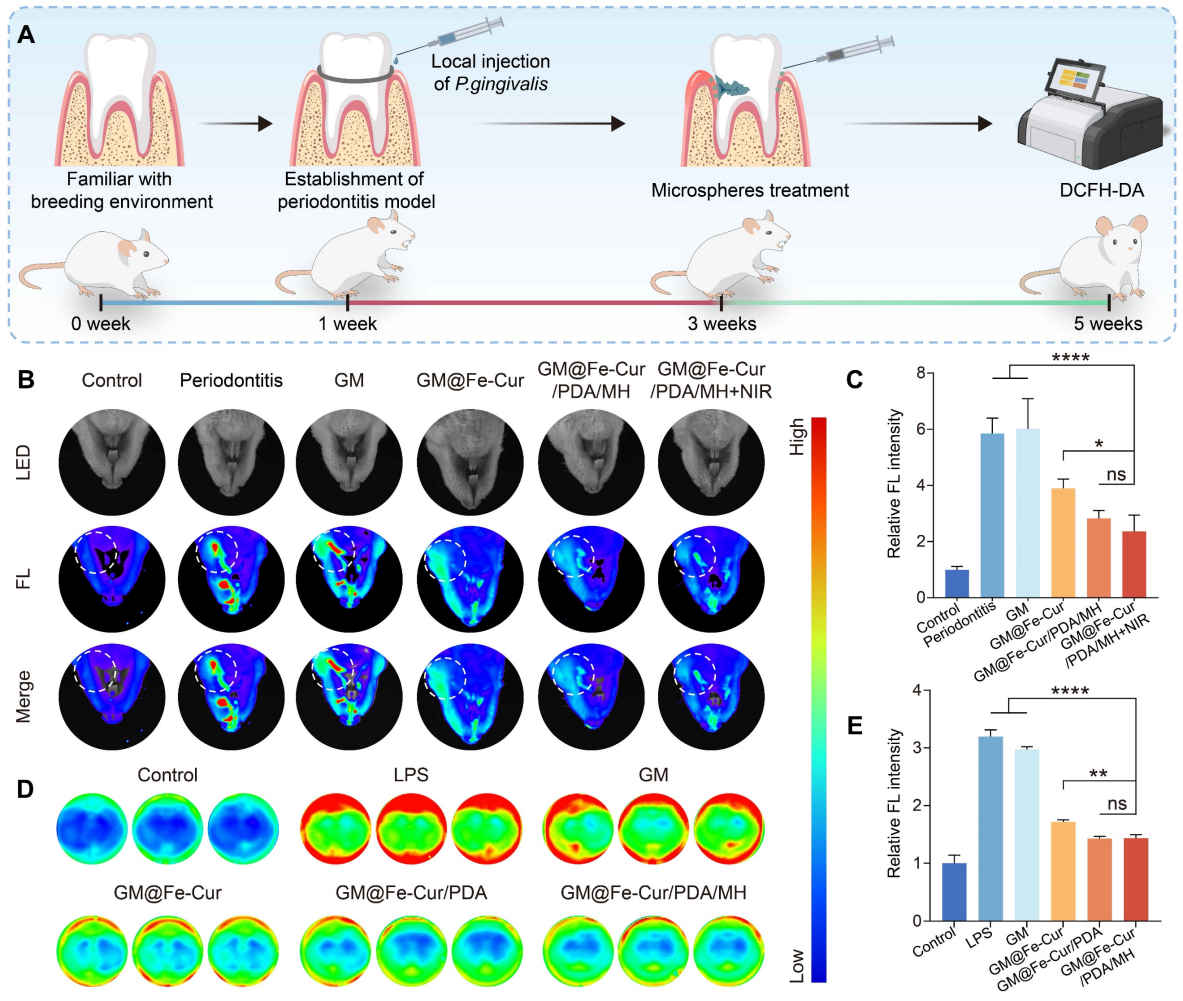
** ROS scavenging properties of GM@Fe-Cur/PDA/MH in a rat periodontitis model. (A)** Schematic illustration of the rat periodontitis model construction process. **(B)** Fluorescence images showing the ROS scavenging effect of microspheres in rats and **(C)** corresponding quantitative analysis of fluorescence intensity. **(D)** Fluorescence images showing the ROS scavenging effect of microspheres in LPS-stimulated macrophages *in vitro* and **(E)** corresponding quantitative analysis of fluorescence intensity.

GM@Fe-Cur/PDA/MH demonstrated excellent ROS scavenging ability *in vitro*. To further evaluate its ROS clearance capability *in vivo*, a rat periodontitis model was established **(Figure 10A and Figure S8)**. After establishing the model, local treatment with the composite microspheres and ROS levels were assessed using DCFH-DA staining. Compared to the control group, the periodontitis group showed significant fluorescence signals around the first maxillary molar, indicating a marked increase in ROS levels. The GM group exhibited similar ROS levels to the periodontitis group, indicating that the GM group has a limited ability to scavenge ROS. In contrast, the GM@Fe-Cur group significantly reduced ROS levels, although some ROS remained. The most notable ROS clearance was observed in the GM@Fe-Cur/PDA/MH group and the GM@Fe-Cur/PDA/MH+NIR group, with both groups showing a substantial decrease in ROS fluorescence intensity and range. This result suggests that the GM@Fe-Cur/PDA/MH+NIR treatment exhibits enhanced ROS scavenging ability **(Figure 10B-C)**.

Additionally, an *in vitro* model was used to stimulate macrophages with LPS to induce ROS production, simulating an inflammatory environment. Following LPS stimulation, intracellular ROS levels in macrophages increased significantly, as evidenced by stronger red fluorescence signals. However, after treatment with GM@Fe-Cur/PDA/MH, the ROS fluorescence intensity decreased markedly, narrowing the gap with the control group **(Figure 10D-E)**. This result aligns with the *in vivo* findings, confirming that GM@Fe-Cur/PDA/MH effectively clears ROS *in vivo* and *in vitro*, supporting its application in periodontitis treatment.

### *In vivo* characterization of GM@Fe-Cur/PDA/MH to alleviate bone resorption

**Figure 11:**
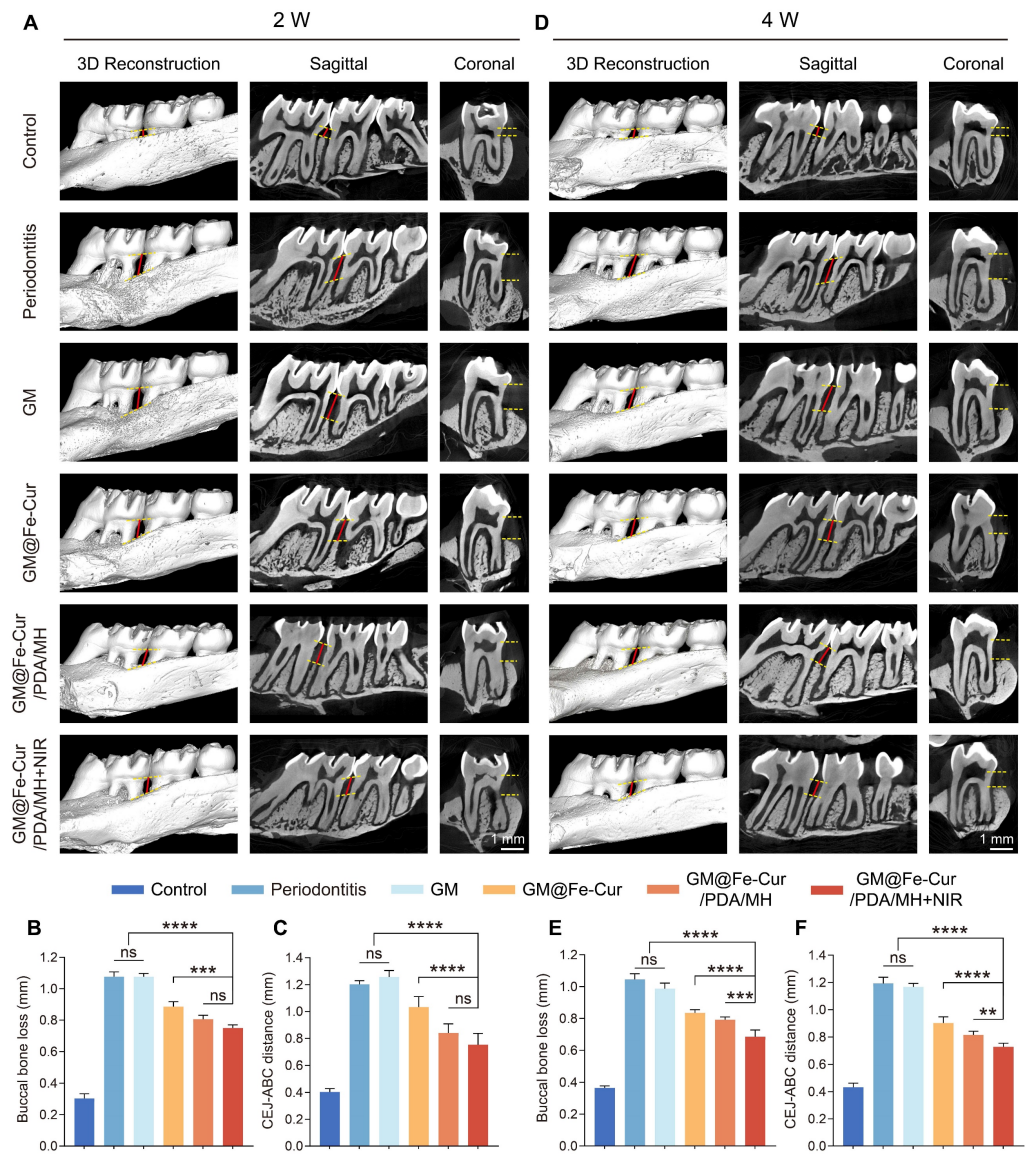
** GM@Fe-Cur/PDA/MH mitigates ligation-induced alveolar bone loss in a rat periodontitis model. (A)** 3D reconstruction, sagittal, and coronal views of periodontal conditions after 2 weeks of microsphere treatment. **(B)** Quantification of buccal bone loss height after 2 weeks. **(C)** CEJ-ABC distance analysis after 2 weeks. **(D)** 3D reconstruction, sagittal, and coronal views after 4 weeks of treatment. **(E)** Quantification of buccal bone loss height after 4 weeks. **(F)** CEJ-ABC distance analysis after 4 weeks.

Under simulated oxidative stress conditions *in vitro*, GM@Fe-Cur/PDA/MH successfully alleviated oxidative damage in hPDLSCs and effectively restored their osteogenic differentiation capacity. Based on these findings, we further conducted *in vivo* evaluations. Maxillary samples were collected post-microsphere treatment in the 2nd and 4th weeks, followed by Micro-CT scanning and analysis. The severity of alveolar bone loss was assessed by measuring the distance from the alveolar bone crest (ABC) at the distal of the first molar to the cemento-enamel junction (CEJ) 52. As shown in **Figure 11A**, the buccal bone defects in the periodontitis group were extensive, with significant alveolar bone resorption and destruction observed at the root furcation. CEJ-ABC measurements indicated that the alveolar bone defect distance exceeded 1.20 mm, whereas the control group showed only about 0.40 mm, confirming the successful establishment of the periodontitis model **(Figure 11B-C)**. At the 4th week of treatment, CEJ-ABC measurements revealed that the GM@Fe-Cur/PDA/MH+NIR group (0.73 ± 0.03 mm) significantly reduced alveolar bone loss caused by periodontal inflammation and restored part of the alveolar bone height, compared with the periodontitis group (1.19 ± 0.05 mm) and the GM group (1.17 ± 0.03 mm) **(Figure 11D-F)**. Additionally, in the 2nd week, there was no significant difference in treatment effects between the GM@Fe-Cur/PDA/MH+NIR group and the GM@Fe-Cur/PDA/MH group. However, in the 4th week, the former demonstrated better therapeutic outcomes, indicating that NIR irradiation resulted in fewer surviving pathogenic bacteria, which contributed to better alleviation of alveolar bone resorption and promoted bone repair and regeneration **(Figure 11C and Figure 11F)**.

In conclusion, the complete treatment of periodontitis requires effective anti-inflammatory, antioxidant, and antimicrobial therapies to relieve the inhibition of bone formation caused by excess ROS, thereby restoring ideal bone height.

### Histological analysis

**Figure 12:**
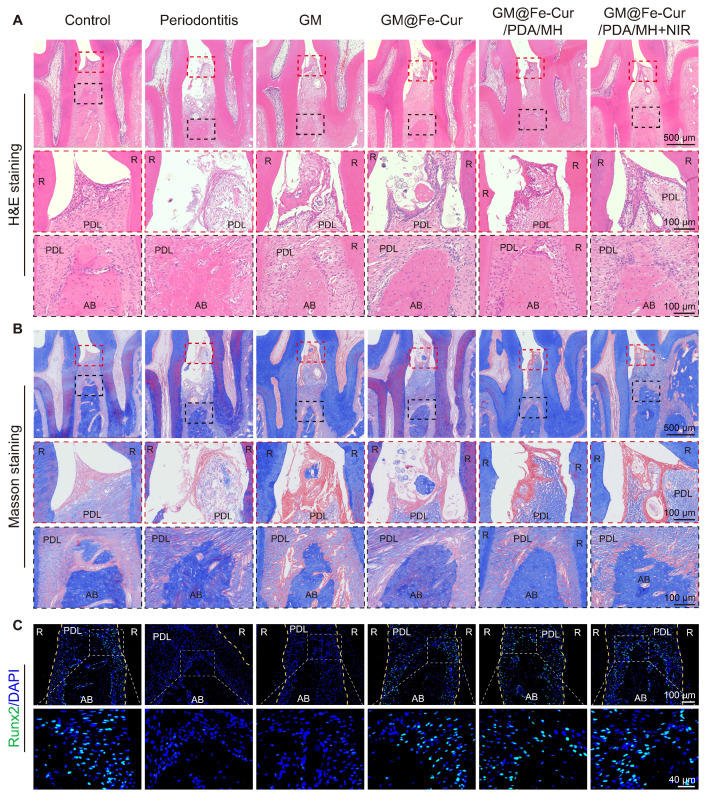
** Evaluation of periodontal soft tissue and bone regeneration after 2 weeks of treatment with different microspheres. (A)** H&E-stained Images. **(B)** Masson's trichrome-stained images. **(C)** Immunofluorescence staining of Runx2. (PDL: periodontal ligament, AB: alveolar bone, R: root).

**Figure 13:**
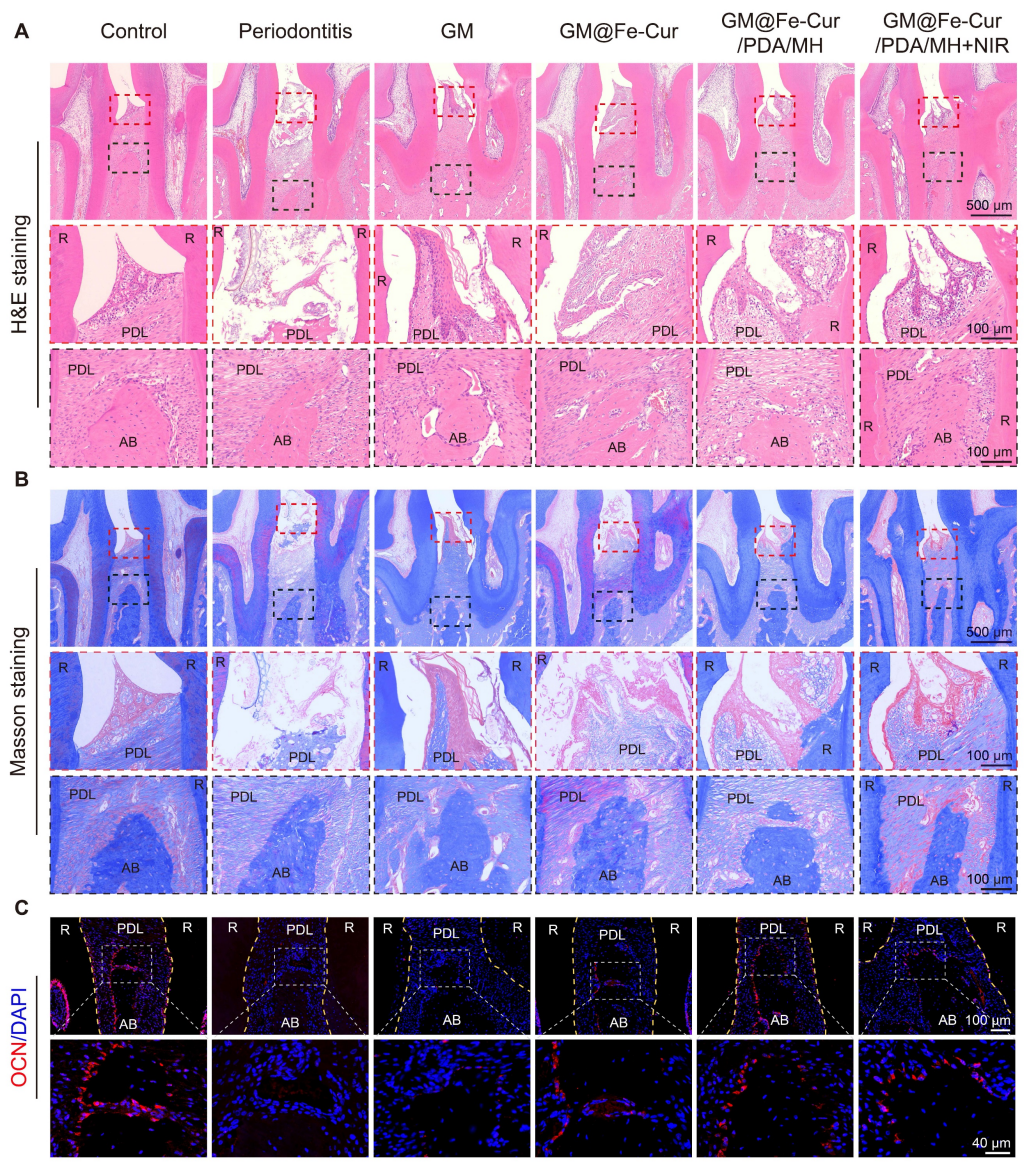
** Evaluation of periodontal soft tissue and bone regeneration after 4 weeks of treatment with different microspheres. (A)** H&E-stained Images. **(B)** Masson's trichrome-stained images. **(C)** Immunofluorescence staining of OCN. (PDL: periodontal ligament, AB: alveolar bone, R: root).

Periodontitis is primarily characterized by the destruction of periodontal soft tissues and the loss of alveolar bone. To assess the impact of GM@Fe-Cur/PDA/MH on periodontal tissues, maxillary tissue sections were observed through H&E and Masson trichrome staining. The staining results at week 2 are shown in **Figure 12A-B**. In the control group, uniform staining, intact gingival epithelial tissue structure, tightly arranged collagen fibers, and normal height and structure of alveolar bone were observed. In contrast, the periodontitis group exhibited significant structural disruption in the gingival epithelial tissue, extensive inflammatory cell infiltration, and marked destruction of alveolar bone height and trabecular structure, indicating severe damage to periodontal tissues 53. The GM group also showed loose epithelial tissue structure, disordered fiber arrangement, and obvious bone resorption, demonstrating a lack of therapeutic effect on periodontitis. In comparison, treatment with GM@Fe-Cur, GM@Fe-Cur/PDA/MH, and GM@Fe-Cur/PDA/MH+NIR resulted in partial restoration of epithelial tissue structure, gradual alignment of fibers, and an increase in alveolar bone height. After 4 weeks of treatment **(Figure 13A-B)**, the collagen fibers in the GM@Fe-Cur/PDA/MH+NIR group became more compact and orderly, with the tissue structure gradually resembling normal conditions, demonstrating significant therapeutic effects on periodontitis.

Additionally, immunofluorescence staining for Runx2 and OCN was used to evaluate osteogenic activity after 2 and 4 weeks of treatment **(Figure 12C and Figure 13C)**. In the control group, the fluorescence staining of these two osteogenesis-related proteins was strong and uniform, whereas, in the periodontitis group and GM group, it appeared dim and weak. In contrast, the expression levels in the GM@Fe-Cur, GM@Fe-Cur/PDA/MH, and GM@Fe-Cur/PDA/MH+NIR groups were significantly elevated, suggesting a restoration of osteogenic activity.

**Figure 14:**
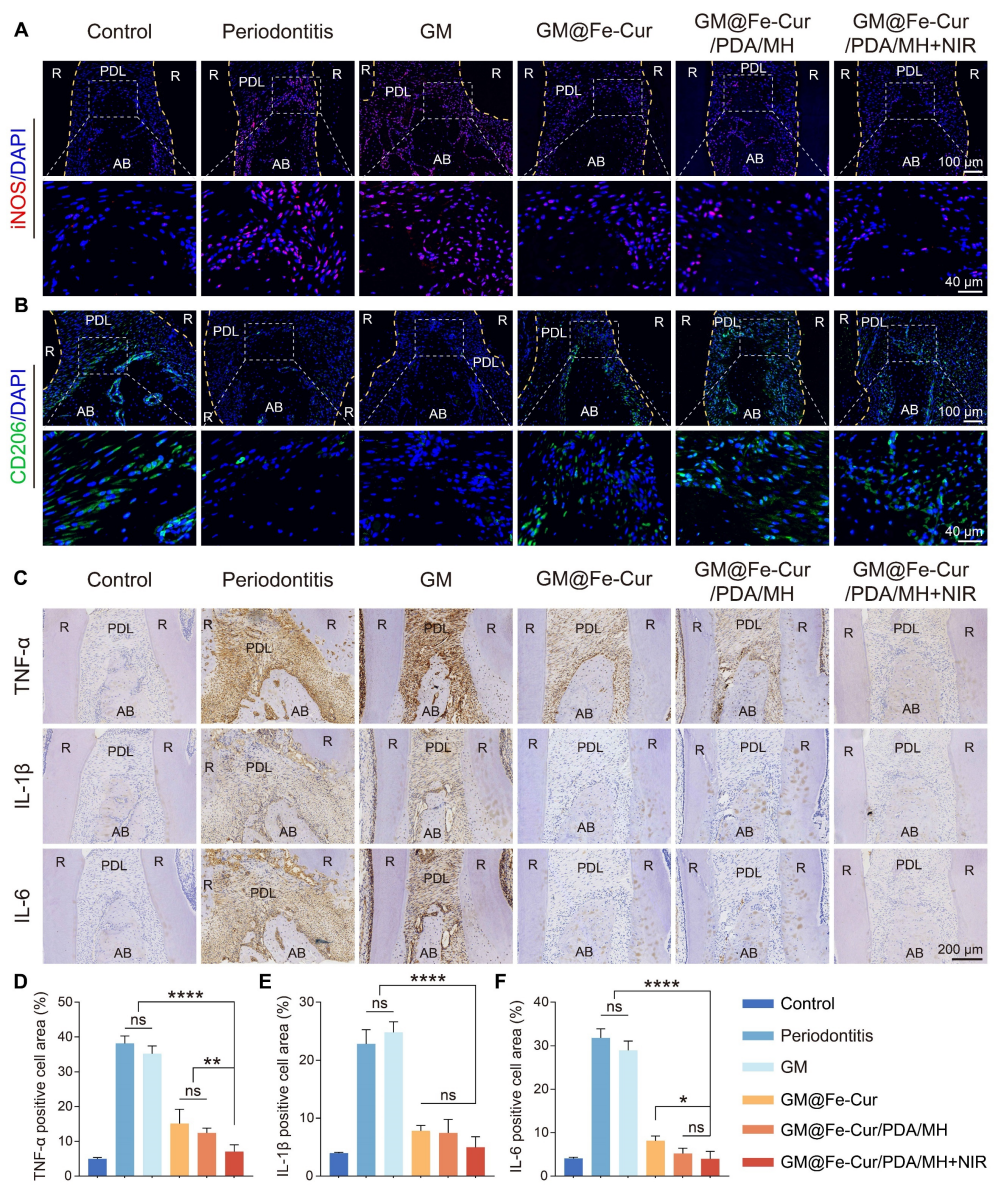
** GM@Fe-Cur/PDA/MH improves the inflammatory status of periodontal tissues in periodontitis rats. (A)** Immunofluorescence staining of iNOS. **(B)** Immunofluorescence staining of CD206. **(C)** Immunohistochemical staining of TNF-α, IL-1β, and IL-6. Percentage area of **(D)** TNF-α, **(E)** IL-1β, and **(F)** IL-6-positive cells in immunohistochemical staining. (PDL: periodontal ligament, AB: alveolar bone, R: root).

To further validate the anti-inflammatory effects of GM@Fe-Cur/PDA/MH, immunofluorescence staining was performed on macrophage surface markers iNOS (M1 type) and CD206 (M2 type), and immunohistochemical staining was conducted for inflammatory cytokines TNF-α, IL-1β, and IL-6. Elevated iNOS expression is commonly associated with various inflammatory diseases and infection states, whereas CD206 expression is typically linked to tissue repair and anti-inflammatory processes 54. The results demonstrated high iNOS expression in the periodontitis and GM groups, indicating a severe inflammatory response **(Figure 14A)**. In contrast, iNOS expression was significantly reduced in the GM@Fe-Cur, GM@Fe-Cur/PDA/MH, and GM@Fe-Cur/PDA/MH+NIR groups, suggesting inflammation was effectively suppressed. On the other hand, CD206 expression was markedly increased in these treatment groups compared to the periodontitis and GM groups, indicating that the tissue repair process was actively progressing **(Figure 14B)**.

TNF-α, IL-1β, and IL-6 play key roles in the inflammatory response and interact within a complex cytokine network under pathological conditions 55. Immunohistochemical staining showed low expression levels of these three inflammatory cytokines in the control group, with almost no visible brown staining regions **(Figure 14C)**. In contrast, the periodontitis and GM groups exhibited extensive brown staining around the alveolar bone, reflecting a vigorous inflammatory response. Treatment with GM@Fe-Cur, GM@Fe-Cur/PDA/MH, and GM@Fe-Cur/PDA/MH+NIR significantly reduced the expression of these inflammatory cytokines, with IL-1β and IL-6 almost absent, indicating that inflammation was effectively suppressed. Quantitative analysis further confirmed these findings **(Figure 14D-F)**. These results suggest that GM@Fe-Cur/PDA/MH effectively inhibited the inflammatory response, gradually shifting periodontal tissues from inflammatory damage to an anti-inflammatory repair state.

### Anti-inflammatory and antioxidant mechanisms of GM@Fe-Cur/PDA/MH

Nrf2 is a critical transcription factor that upregulates the expression of various antioxidant genes, including heme oxygenase-1 (HO-1) and NAD(P)H quinone dehydrogenase 1 (NQO-1) 26. Under homeostatic conditions, Nrf2 primarily binds to Keap1 (Kelch-like ECH-associated protein 1) and is subsequently degraded *via* the ubiquitin-proteasome pathway. When cells are exposed to oxidative stress or other stimuli, Nrf2 is released from Keap1 and translocates to the nucleus, where it initiates the expression of antioxidant genes 56. Activation of the Nrf2 signaling pathway has shown significant protective effects in various inflammatory diseases, and recent research increasingly highlights Nrf2's crucial role in the progression of periodontitis 57. NLRP3 inflammasome is a multiprotein complex. When stimulated by LPS, the NLRP3 receptor activates Caspase-1, forming an intact inflammasome complex 58. Caspase-1 activation triggers the maturation of potent pro-inflammatory cytokines, such as IL-1β and IL-18, which amplify the immune response 59. Our experimental results indicate that GM@Fe-Cur/PDA/MH inhibits macrophage polarization toward the M1 phenotype and promotes polarization toward the M2 phenotype, accompanied by downregulation of pro-inflammatory genes (TNF-α and IL-1β) and upregulation of anti-inflammatory genes (Arg-1 and IL-10). Based on these findings, we hypothesize that GM@Fe-Cur/PDA/MH may exert its anti-inflammatory effects by activating the Nrf2 signaling pathway and inhibiting the assembly of the NLRP3 inflammasome.

**Figure 15:**
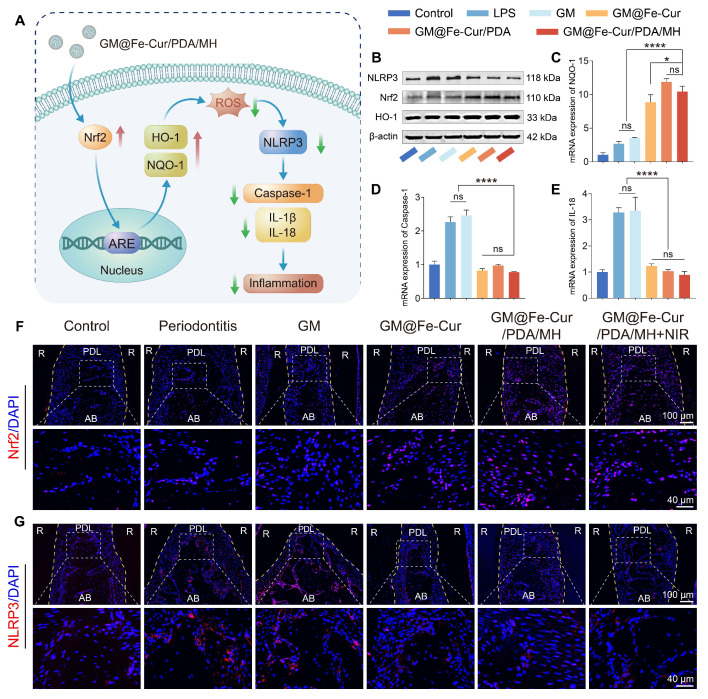
** Investigation of the anti-inflammatory and antioxidant mechanisms of GM@Fe-Cur/PDA/MH. (A)** Schematic representation of the antioxidant and anti-inflammatory mechanisms of GM@Fe-Cur/PDA/MH. **(B)** Western blot analysis of NLRP3, Nrf2, and HO-1, with β-actin as the internal control. **(C)** NQO-1 mRNA expression *in vitro*. **(D)** Caspase-1 mRNA expression *in vitro*. **(E)** IL-18 mRNA expression *in vitro*. Immunofluorescence staining of **(F)** Nrf2 and **(G)** NLRP3 *in vivo*. (PDL: periodontal ligament, AB: alveolar bone, R: root).

To verify this hypothesis, we evaluated the protein expression levels of Nrf2, HO-1, and NLRP3 and the gene expression levels of NQO-1, Caspase-1, and IL-18 using Western blot and qRT-PCR. The results showed that, following LPS stimulation, the protein expression of Nrf2 and HO-1 and the gene expression of NQO-1 were elevated. GM@Fe-Cur/PDA/MH treatment further amplified this trend, indicating significant activation of Nrf2 and its downstream antioxidant genes HO-1 and NQO-1 **(Figure 15B-C)**. Compared to the control group, the LPS-stimulated group showed a significant increase in NLRP3 protein expression and Caspase-1 and IL-18 gene expression, while GM@Fe-Cur/PDA/MH treatment effectively inhibited these changes **(Figure 15D-E)**.

Next, we used immunofluorescence staining to examine the protein expression levels of Nrf2 and NLRP3 in periodontal tissues. As shown in **Figure 15F**, Nrf2 expression was increased in the periodontitis group, likely due to the oxidative stress induced by the inflammatory microenvironment. After GM@Fe-Cur/PDA/MH treatment, the fluorescence intensity increased significantly, indicating that the expression of Nrf2 was further enhanced. Immunofluorescence results for NLRP3 showed that its expression was significantly higher in the periodontitis group compared to the control group, while GM@Fe-Cur/PDA/MH treatment effectively suppressed the excessive expression of NLRP3 **(Figure 15G)**.

In summary, these data suggest that GM@Fe-Cur/PDA/MH promotes the expression of Nrf2 and its downstream antioxidant genes NQO-1 and HO-1, enhancing cellular antioxidant capacity while inhibiting the release of pro-inflammatory factors such as Caspase-1, IL-1β, and IL-18. This property reduces the inflammatory response and improves the inflammatory microenvironment in periodontal tissues. Thus, the regulatory mechanism of GM@Fe-Cur/PDA/MH is significant in controlling inflammation and reducing tissue damage, offering potential therapeutic value for treating periodontitis.

## Conclusion

In summary, we successfully synthesized fully water-soluble Fe-Cur NPs by utilizing iron ion-coordinated curcumin. By encapsulating these nanoparticles within GM and leveraging the multifunctional properties of PDA, we introduced photothermal responsiveness and antimicrobial properties, creating GM@Fe-Cur/PDA/MH. *In vitro* studies demonstrated that GM@Fe-Cur/PDA/MH possesses outstanding ROS scavenging abilities, mitigating oxidative stress-induced cellular damage and restoring cellular morphology and osteogenic functions. The combined antimicrobial activity of MH and the photothermal effect of PDA exhibited superior synergistic effects in inhibiting the growth of various bacteria. Additionally, GM@Fe-Cur/PDA/MH exerts immunomodulatory effects by inhibiting M1 macrophage polarization and promoting M2 polarization, thereby reducing the secretion of pro-inflammatory cytokines and increasing the expression of anti-inflammatory cytokines. Further studies in a rat periodontitis model revealed that GM@Fe-Cur/PDA/MH effectively scavenges ROS *in vivo*, alleviates inflammatory responses, and reduces alveolar bone resorption. Our research also indicates that GM@Fe-Cur/PDA/MH protects cells from oxidative damage and inflammatory environments by activating the Nrf2 signaling pathway and inhibiting the activation of the NLRP3 inflammasome. In conclusion, through the synergistic effects of multiple mechanisms, GM@Fe-Cur/PDA/MH offers a promising and innovative therapeutic strategy for treating periodontitis with significant potential for clinical application.

## Supplementary Material

Supplementary figures and table.
