# Supplementary material for: A multifunctional injectable microsphere with enhanced near-infrared photo-antibacterial, ROS scavenging, and anti-inflammatory properties for periodontitis treatment
Source: Theranostics. 2025 Mar 3;15(9):3750–80. doi: 10.7150/thno.107793 (PMC11980662; doi:10.7150/thno.107793)
Supplement: Supplementary file 1 — Supplementary figures and table. [file thnov15p3750s1.pdf]

A multifunctional injectable microsphere with enhanced near-infrared photo-antibacterial, ROS scavenging, and anti-inflammatory properties for periodontitis treatment

Ziliang Xiu[1, 2], Yujing Zhu[1, 2], Xiaofeng Li[1, 2], Xiaoxi Jiang[3], Yunru Feng[1, 2], Li He[1, 2], Chunhui Li[1, 2, 4*], Rui Cai[1, 4*], Gang Tao[1, 4*]

[1] Oral & Maxillofacial Reconstruction and Regeneration of Luzhou Key Laboratory, The Affiliated Stomatological Hospital, Southwest Medical University, Luzhou 646000, China

[2] Department of Periodontics & Oral Mucosal Diseases, The Affiliated Stomatological Hospital, Southwest Medical University, Luzhou 646000, China.

[3] Department of Oral Medicine, Deyang Stomatological Hospital, Deyang 618000, China.

[4] Institute of Stomatology, Southwest Medical University, Luzhou 646000, China

*Correspondence:

Chunhui Li, Oral & Maxillofacial Reconstruction and Regeneration of Luzhou Key Laboratory, The Affiliated Stomatological Hospital, Southwest Medical University, Luzhou 646000, China, Email: lch10221022@163.com

Rui Cai, Oral & Maxillofacial Reconstruction and Regeneration of Luzhou Key Laboratory, The Affiliated Stomatological Hospital, Southwest Medical University, Luzhou 646000, China, E-mail: cairui@swmu.edu.cn

Gang Tao, Oral & Maxillofacial Reconstruction and Regeneration of Luzhou Key Laboratory, The Affiliated Stomatological Hospital, Southwest Medical University, Luzhou 646000, China, E-mail: taogang@swmu.edu.cn

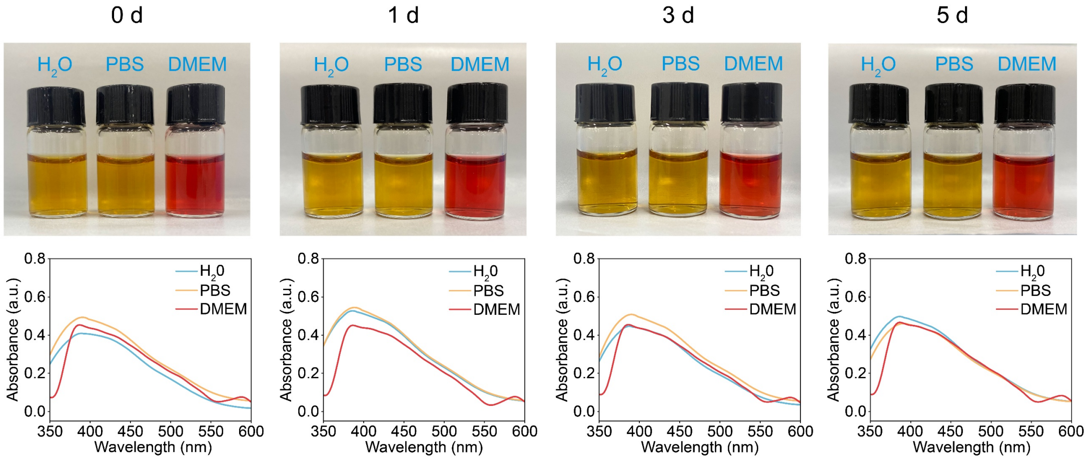

Figure S1. Photographs of Fe-Cur NPs in H_2O, PBS, and DMEM medium for 5 days and the corresponding UV-vis spectra.

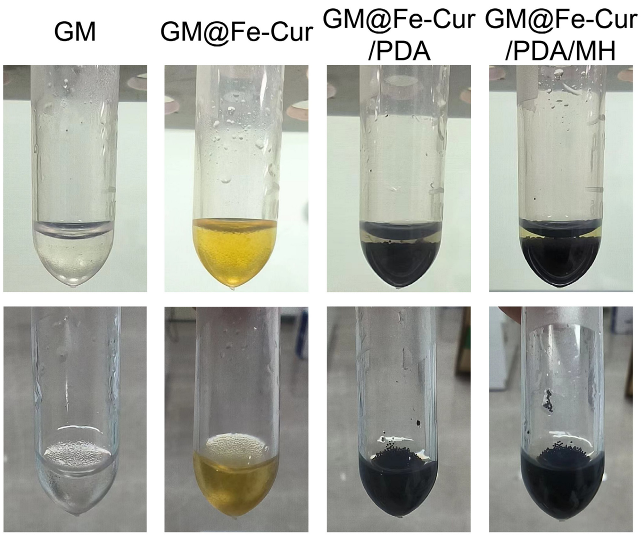

Figure S2. Overall views of four microspheres.

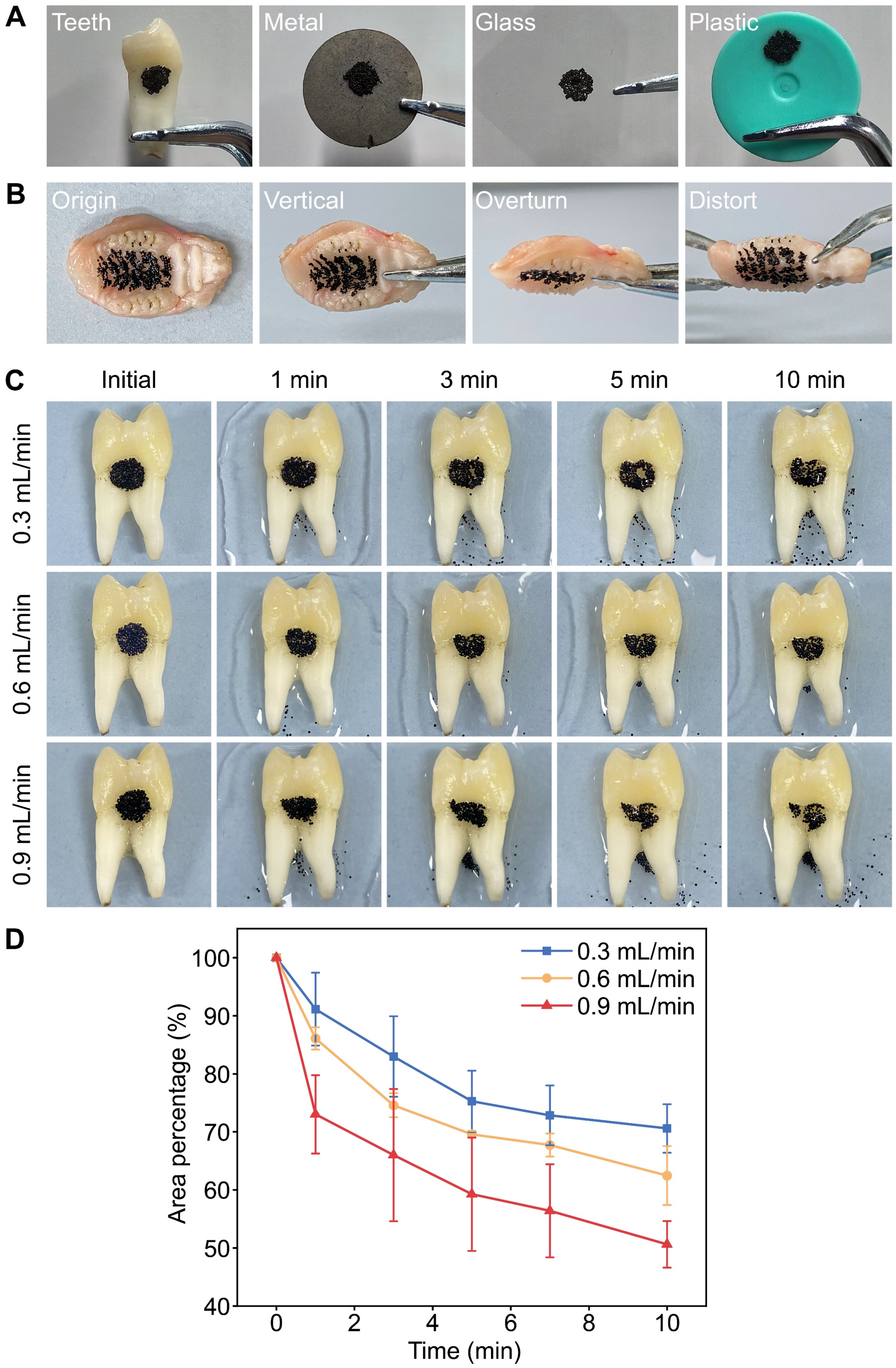

Figure S3. The adhesion test of the GM@Fe-Cur/PDA/MH. (A) Photographs of GM@Fe-Cur/PDA/MH adhered on different materials, such as teeth, metal, glass, and plastic. **(B)** Photographs of the GM@Fe-Cur/PDA/MH adhered on the isolated maxillary gingival tissue of rat before and after overturning and

twisting. **(C)** The Area of retained GM@Fe-Cur/PDA/MH after being subjected to different flow rates over a period of time. **(D)** Quantitative analysis of residual GM@Fe-Cur/PDA/MH area.

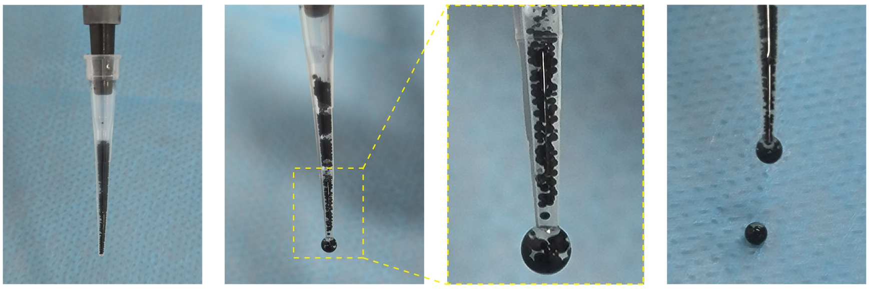

Figure S4. The injectability of GM@Fe-Cur/PDA/MH.

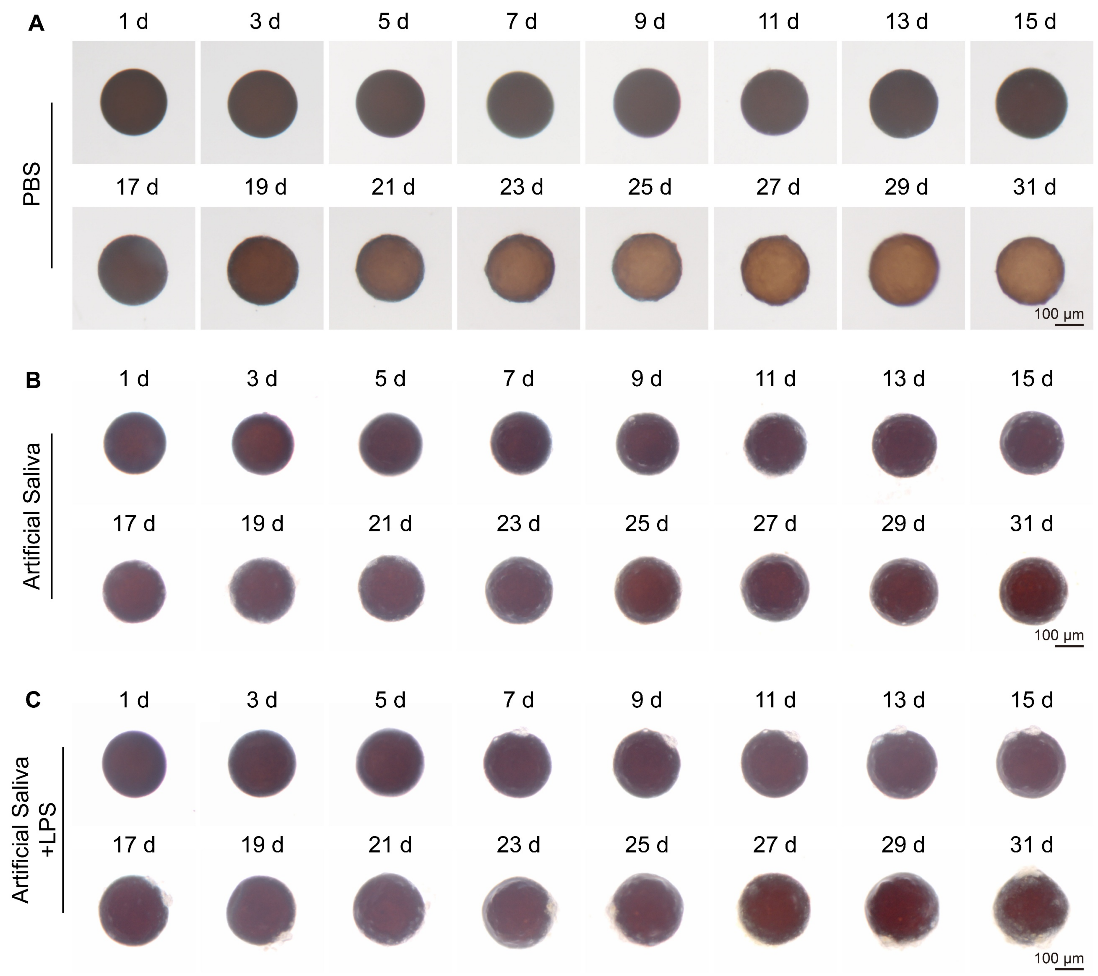

Figure S5. Degradation test of GM@Fe-Cur/PDA/MH. Morphological changes of GM@Fe-Cur/PDA/MH were soaked in different solution media for one month: **(A)** PBS, **(B)** Artificial saliva, and **(C)** Artificial saliva + LPS.

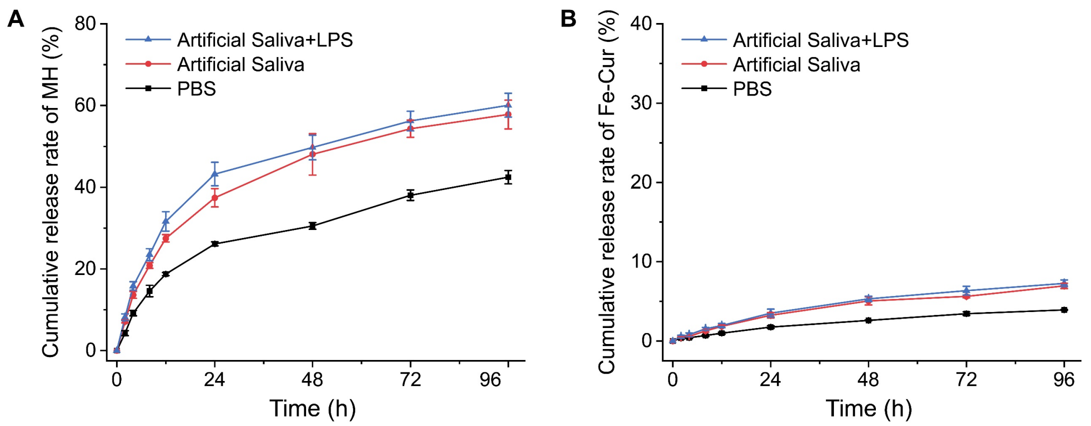

Figure S6. Drug release studies of GM@Fe-Cur/PDA/MH. (A) Cumulative release of MH from GM@Fe-Cur/PDA/MH in the three environments. **(B)** Cumulative release of Fe-Cur NPs from GM@Fe-Cur/PDA/MH in the three environments.

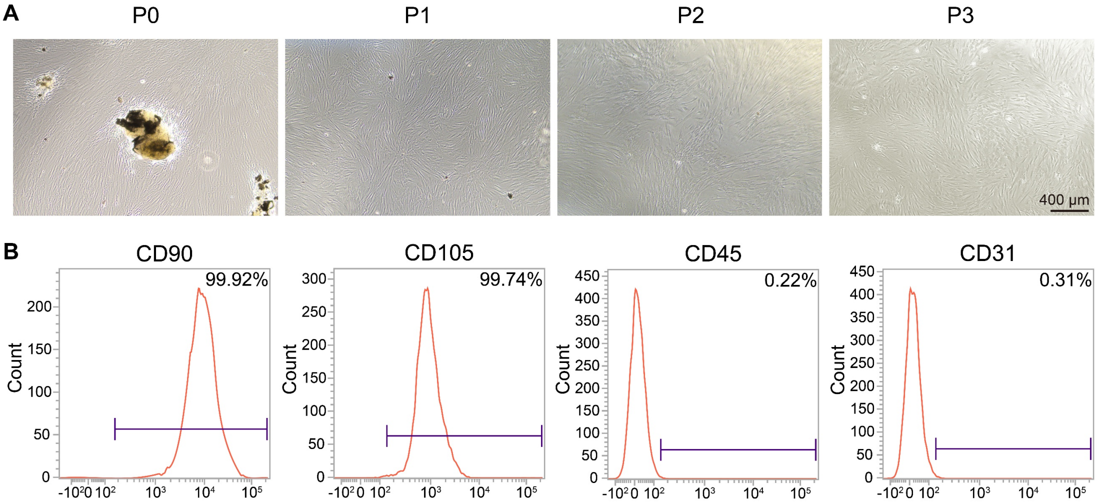

Figure S7. Culture and identification of hPDLSCs. (A) Morphology of hPDLSCs. **(B)** Flow cytometric analysis of cell surface markers, including the expression of CD90, CD105, CD45, and CD31 in hPDLSCs.

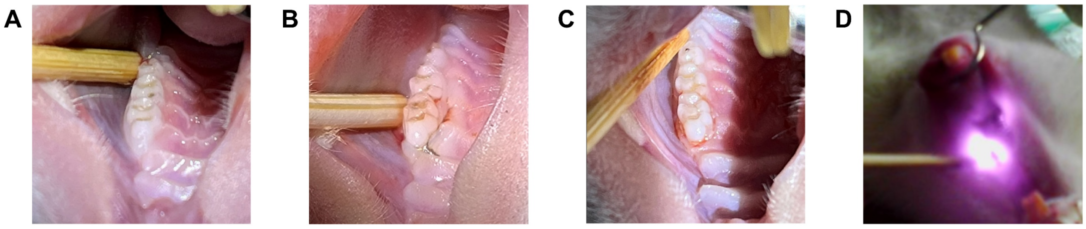

Figure S8. Schematic representation of the animal experimental treatment protocol. (A) Healthy periodontal tissue. **(B)** Placement of ligature wire on the maxillary first molar and injection of bacterial suspension. **(C)** Removal of the ligature wire after 2 weeks of local ligation. **(D)** Irradiation of the ligation site with 808 nm NIR light.

Table S1. Primer sequences used in qRT-PCR study.

Gene	Primer Sequence (5'-3')
Caspase-1	F: TATCCAGGAGGGAATATGTG<br>R: ACAACACCACTCCTTGTTTC
NQO-1	F: CGCCTGAGCCCAGATATTGT<br>R: ACCACTGCAATGGGAACTGA
IL-18	F: AGTAAGAGGACTGGCTGTGACC<br>R: TTGGCAAGCAAGAAAGTGTC
TNF-α	F: CGCTGAGGTCAATCTGC<br>R: GGCTGGGTAGAGAATGGA
IL-1β	F: TGGTGTGTGACGTTCCC<br>R: TGTCCATTGAGGTGGAGAG
IL-10	F: GCCAGAGCCACATGCTCCTA<br>R: GTCCAGCTGGTCCTTTGTTTG
Arg-1	F: GGGCTCCTTTCAGGACTAGATA<br>R: CGAAGCAAGCCAAGGTTAAAG
β-actin	F: GGCTGTATTCCCCTCCATCG<br>R: CCAGTTGGTAACAATGCCATGT
